# From blueprint to build: Metal ions in peripheral nerve development and engineering regeneration

**DOI:** 10.1016/j.bioactmat.2026.05.019

**Published:** 2026-06-09

**Authors:** Mouyuan Sun, Yaxian Luo, Zhixu He, Yan Tu, Shuangyang Li, Luying Qin, Jingyu Zhang, Lianjie Peng, Tao Qiu, Tian Zhang, Huiming Wang, Haifei Shi, Yong He, Mengfei Yu

**Affiliations:** a Stomatology Hospital, School of Stomatology, Zhejiang University School of Medicine, Zhejiang Provincial Clinical Research Center for Oral Diseases, Zhejiang Key Laboratory of Oral Biomedical, Hangzhou, 310000, China; b State Key Laboratory of Fluid Power and Mechatronic Systems, School of Mechanical Engineering, Key Laboratory of 3D Printing Process and Equipment of Zhejiang Province, Zhejiang University, Hangzhou, 310027, China; c Department of Hand Surgery, First Affiliated Hospital of Zhejiang University, School of Medicine, Hangzhou, 310003, China

**Keywords:** Metal ions, Peripheral nerve development, Peripheral nerve regeneration, scRNA-seq, Development - inspired tissue engineering scaffolds

## Abstract

Peripheral nerve injury (PNI) poses a substantial global health burden, affecting over 20 million individuals annually with frequent suboptimal recovery and persistent disability. The inherent limitations of nerve autografts, such as donor site morbidity and limited supply, underscore the clinical importance of nerve guidance conduits (NGCs) as a promising alternative. Nonetheless, the efficacy of current NGCs remains limited by their inability to recapitulate the spatiotemporally precise molecular cues of the native regenerative microenvironment. Our network meta-analysis highlights a significant efficacy gap in current NGCs and underscores the urgent clinical need for novel bioactive strategies. Metal ions have emerged as pivotal therapeutic candidates, capable of orchestrating regeneration by reactivating developmental programs through their tunable release kinetics and pleiotropic effects. By integrating spatiotemporal metal ion dynamics with single-cell transcriptomic data, this review deciphers a conserved, cell-type-specific signaling axis that operates across both development and regeneration. The review further evaluates advanced tissue engineering platforms, ranging from biodegradable metals to functional polymers, coupled with innovative fabrication technologies that enable spatiotemporally controlled ion delivery. This synthesis culminates in a development-inspired engineering blueprint that proposes a paradigm shift from passive structural support to active, development-mimetic instruction, ultimately aiming to accelerate the clinical translation of metal ion–based therapies for PNI (Scheme 1).

## Highlights

• Clinical evidence reveals limited nerve conduit efficacy, demanding bioactive repair strategies.
• Elucidates metal-ion spatiotemporal dynamics by integrating single-cell analysis.
• Proposes a development-inspired paradigm leveraging metal ions as instructive bioactive cues.
• Provides a translational roadmap from advanced biomaterials to clinical application.

## Graphical abstract

**Figure:**
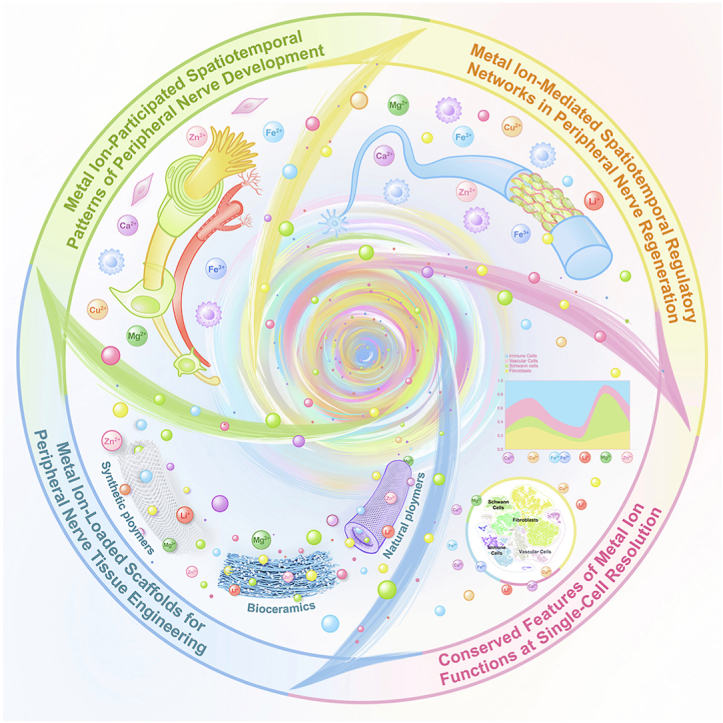

## 1 Introduction

**Scheme 1:**
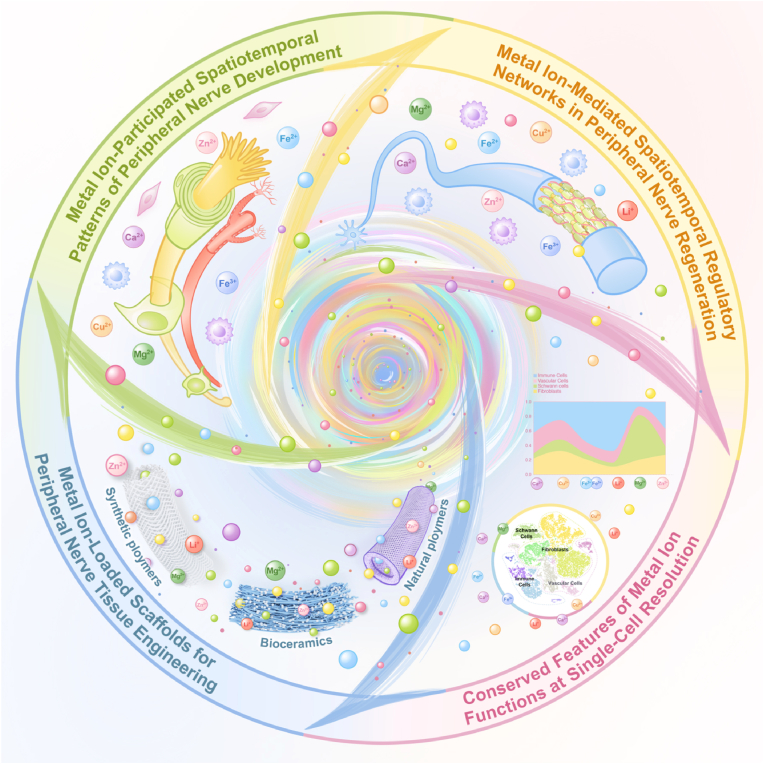
Highlighting a novel “metal ion storm” paradigm, this graphical abstract depicts its pivotal role in peripheral nerve regeneration. A meticulously coordinated storm of metal ions is shown directing the ordered regeneration of nerves, adhering to spatiotemporal principles derived from developmental biology. This imagery underscores the ions' precise, cell-type-specific regulatory functions. The composition further merges a single-cell-based regulatory network with an engineered nerve conduit, exemplifying the translation from mechanistic insight to therapeutic design. The overall visual narrative powerfully conveys how these ion-based signals create an instructive microenvironment, shifting the treatment strategy from passive scaffolding to active biological instruction.

The peripheral nervous system (PNS) constitutes a critical structural interface, connecting the central nervous system with peripheral organs to enable essential motor and sensory functions [1,2]. However, its inherently limited regenerative capacity renders it vulnerable to trauma, disease, or injury, frequently leading to substantial functional deficits. Peripheral nerve injury (PNI) affects over 20 million patients in both the United States and China annually, often causing impaired motor and sensory performance [3,4]. A substantial proportion of these patients endure long-term complications, including permanent disability and chronic pain, which severely diminish quality of life and impose considerable socioeconomic burdens [4]. Current clinical interventions for PNI yield suboptimal outcomes, despite continued refinements in surgical techniques. Direct end-to-end neurorrhaphy, for instance, carries a risk of postoperative inflammation and is ineffective for nerve gaps exceeding 2 cm, as mechanical tension impedes vascularization and axonal regeneration [5]. While autologous nerve transplantation remains the clinical gold standard, its application is limited by donor site morbidity, scarce graft availability, and potential immune rejection [6]. As an alternative, nerve guidance conduits (NGCs) provide physical scaffolding to support nerve regrowth, yet currently approved versions lack the necessary bioactive cues to effectively direct the regenerative process, resulting in unsatisfactory functional recovery [7,8]. Consequently, fewer than 50% of patients regain satisfactory function post-intervention [9]. The rising global incidence of PNI, compounded by the limitations of existing therapeutic options, underscores an urgent imperative to elucidate the fundamental mechanisms of nerve regeneration and to develop advanced biomimetic strategies that recapitulate the spatiotemporal complexity of the native repair process (see Scheme 1).

Upon PNI, a precisely orchestrated regenerative sequence is initiated. This begins with Wallerian degeneration at the lesion site, involving the breakdown of distal axons and myelin. Subsequently, activated Schwann cells dedifferentiate, proliferate, and migrate, collaborating with macrophages to clear cellular debris. Concurrently, they organize into Büngner bands and secrete neurotrophic factors that direct the regrowth of proximal axons into the distal stump, ultimately facilitating functional restoration [10,11]. This intricate cascade is critically modulated by specific metal ions: lithium and calcium remodel growth cones to propel axonal extension; magnesium and lithium drive Schwann cell dedifferentiation; magnesium and iron fine-tune the immune milieu; and zinc and copper foster angiogenesis [[12], [13], [14]]. Critically, these metal ions operate as an integrated regulatory network, exhibiting concentration-dependent effects and temporal specificity that mirror the phased progression of nerve regeneration. Their evolutionarily conserved roles, echoing those in development, create a molecular link connecting ontogenetic and repair pathways. Specifically, calcium directs axon guidance, whereas magnesium, zinc, iron, and copper jointly shape a supportive microenvironment [[15], [16], [17], [18]]. Such functional conservation aligns with the fundamental principle in regeneration biology that tissue repair often recapitulates developmental processes [19,20]. Within this conceptual framework, metal ions emerge as pivotal coordinators, synchronizing developmental cues with repair mechanisms. This conserved functionality provides a strategic blueprint for biomimetic interventions that target ion-modulated pathways to enhance nerve repair. Nonetheless, the spatially and temporally precise delivery of these ions to the injury site remains a central challenge for clinical translation.

Emerging manufacturing technologies are advancing the sophisticated integration of metal ions into functional NGCs. Innovations in material composites, nanoparticle incorporation, and surface functionalization are shifting the paradigm from single-ion release toward multi-ionic synergy and spatiotemporally controlled delivery. However, each method presents specific limitations: material composites may exhibit interfacial incompatibility that undermines structural integrity [21]; nanoparticle systems, despite their bioactivity, often confront issues of aggregation and heterogeneous distribution [22]; and surface functionalization can be constrained by unstable adhesion or insufficient binding specificity [23]. Beyond these material and processing hurdles, the intrinsic biological complexity of metal ions presents a further therapeutic challenge. Crucial considerations include divergent concentration thresholds, dynamic ion-ion interactions, and phase-specific functional requirements, all of which necessitate a precise alignment of ion combinations, release kinetics, and spatial positioning with the native regenerative sequence [24]. Thus, deciphering the coordinated regulation of metal ions throughout the regenerative cascade, from initial inflammation to functional reinnervation, is of critical importance. Adopting this holistic view will facilitate the optimization of current delivery strategies and inform the design of next-generation scaffolds that more faithfully emulate the sophisticated biological program of native regeneration.

Guided by a network meta-analysis that underscores the limitations of current NGCs, this review articulates a comprehensive translational roadmap for metal ion-based therapies in peripheral nerve regeneration (PNR). It systematically examines the spatiotemporal regulatory functions of metal ions, synthesizing evidence on their cellular dynamics, concentration-dependent effects, and molecular mechanisms across developmental and regenerative contexts. Building on this mechanistic foundation, the review critically evaluates advanced tissue engineering platforms for ion delivery, covering metal implants, natural and synthetic polymer scaffolds, and bioceramics, in conjunction with enabling fabrication technologies such as electrospinning, freeze-drying, and 3D printing. In its concluding outlook, the review identifies key translational challenges and emerging opportunities, highlighting the potential of development-inspired, metal ion-integrated strategies to bridge the gap between fundamental neurobiology and clinically viable solutions for PNR.

## 2 Meta-analysis reveals suboptimal efficacy of current nerve guidance conduits

**Fig. 1:**
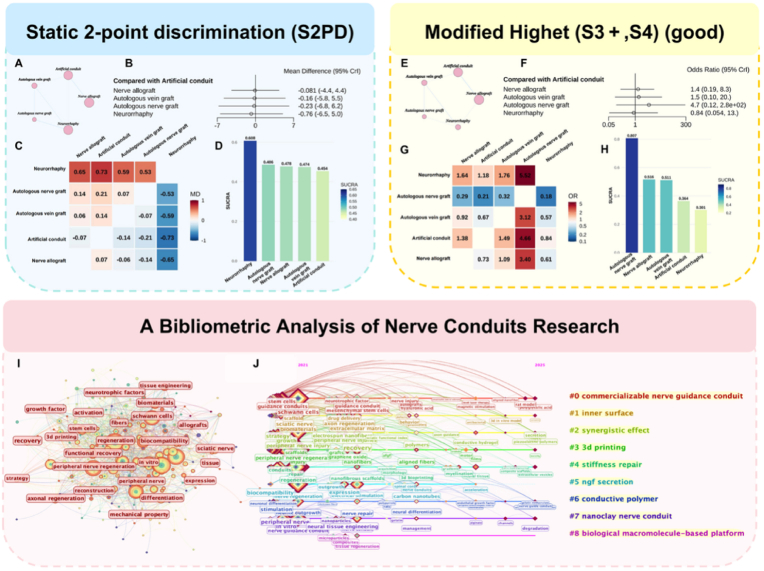
**Clinical Efficacy Landscape of Current Nerve Conduits: Evidence from Network Meta-Analysis and Bibliometric Analysis.** (A-D) Network meta-analysis for the outcome of static two-point discrimination (S2PD): (A) Network geometry of available treatment comparisons. Each node represents an intervention, and the width of the lines connecting them is proportional to the number of direct comparisons; (B) Forest plot displaying the mean differences (MD) and 95% credible intervals (CrI) of each intervention compared to the reference treatment; (C) League table presenting pairwise comparisons between all interventions; (D) Surface Under the Cumulative Ranking curve (SUCRA) probabilities for each intervention. A higher SUCRA value indicates a greater likelihood of being the most effective treatment. (E-H) Network meta-analysis for the outcome of functional recovery based on the modified Highet classification: (E) Network geometry of treatment comparisons; (F) Forest plot displaying the odds ratios (OR) and 95% CrI for each intervention compared to the reference; (G) League table for pairwise comparisons; (H) SUCRA probabilities for functional recovery. (I) Keyword co-occurrence network map. (J) Keyword clustering analysis.

Our systematic search encompassed four major databases (PubMed/MEDLINE, Embase, Web of Science, and Cochrane CENTRAL) identifying 68 eligible studies for this analysis (Table 1). We performed a network meta-analysis comparing five peripheral nerve repair techniques: nerve allograft, artificial conduit, autologous vein graft, autologous nerve graft, and neurorrhaphy. Outcomes included static two-point discrimination (S2PD) and the modified Highet classification of nerve recovery (Fig. 1A–E, Supplementary Fig. 1). The results show that the artificial conduit, an emerging minimally invasive technology, did not demonstrate superior efficacy over conventional approaches. League table analysis confirmed that all mean differences and odds ratios comparing the artificial conduit to other interventions had 95% credible intervals crossing the null values (0 or 1), indicating no statistically significant differences (Fig. 1B, C, F, G). The surface under the cumulative ranking curve (SUCRA) metric, which ranges from 0 to 1, represents the probability of an intervention being the optimal treatment. For S2PD improvement, the artificial conduit attained a SUCRA value of only 0.454 (ranking fourth among the five interventions), which further declined to 0.364 for functional recovery according to the Highet classification (Fig. 1D–H). SUCRA values below 0.5 in both outcomes reflect a less than 50% probability of being the optimal choice. These findings suggest that, despite theoretical advantages such as avoiding donor-site morbidity, current artificial nerve conduits have not yet exceeded conventional methods in functional recovery and may even underperform in certain metrics. This highlights the urgent need to develop next-generation conduits that address these clinical limitations.

**Table 1:** The characteristics of included studies.

Study	Study type	Intervention (s) and control	Number of digital nerve repairs analyzed	Never gap mean (range)	Follow-up time mean (range)	Static 2-point discrimination	Modified Highet (S3 + ,S4) (good)
Arnaout 2014 [25]	Prospective	Artificial conduit	24	-	6mth	10.3 mm	85%
Lohmeyer 2009 [26]	Prospective	Artificial conduit	12	12.5 ± 3.7 mm(6 -18 mm)	12mth	9.33 mm(4-15 mm)	75.0%
Isaacs 2023 [27]	Prospective	Nerve allograft VS Artificial conduit; A/B	68(A:35; B:33)	15-25 mm	6-15mth	A: 6.1 ± 3.3 mm	A: 68.6%
						B: 7.5 ± 2.4 mm	B: 63.7%
Kusuhara 2019 [28]	Prospective	Artificial conduit	20	16.7 mm (1–50 mm)	13mth	8.6 ± 1.2 mm	18 (90%)
Mackinnon 1990 [29]	Retrospective	Artificial conduit	15	1.7 cm (0.5–3.0 cm)	22.4 mth (11–32 mth)	4.6 ± 1.1 mm	8 (53%)
Rinker 2011 [30]	Prospective	Artificial conduit VS Autologous vein graft; (A/B)	56 (A:34; B:22)	10 mm (4–25 mm)	A:12 mth; B:12 mth	A:7.5 ± 1.9 mm B:7.6 ± 2.6 mm	-
Battiston 2005 [31]	Retrospective	Artificial conduit VS Autologous vein graft; A/B	32 (A:19; B:13)	A:2 cm (1–4 cm); B:1.1 cm (0.5–1.5 cm)	6–74 mth	-	A:13 (68%) B:10 (77%)
Neubrech 2016 [32]	Retrospective	Artificial conduit VS Neurorrhaphy; A/B	38 (A:15; B:23	2–3 cm	34 mth (10-76 mth)	A: 5.5 mm (3–15; SD: 5) B: 4.5 mm (3–15; SD: 3.9)	-
Bushnell 2008 [33]	Retrospective	Artificial conduit	9	10–20 mm	15 mth (12–22 mth)	6.9 ± 2.9 mm	4 (44%)
Lohmeyer 2014 [34]	Prospective	Artificial conduit	40	12 mm (5–25 mm)	12 mth	-	20 (50%)
Lohmeyer 2007 [35]	Prospective	Artificial conduit	6	18 mm	12 mth	8.3 ± 5.3 mm	5 (83%)
Schmauss 2014 [36]	Prospective	Artificial conduit	20	11.0 mm (6–25 mm)	58.1 mth (29.3–93.3 mth)	6.8 mm (3–15 mm)	17 (85%)
Taras 2011 [37]	Prospective	Artificial conduit	22	12 mm (5–17 mm)	20 mth (12–59 mth)	5.2 ± 1.5 mm	-
Arnaout 2014 [25]	Prospective	Artificial conduit	27	-	6 mth	10.3 ± 3.76 mm	23 (85%)
Thomsen 2010 [38]	Retrospective	Artificial conduit	11	11.25 mm (5–20 mm)	11.8 mth (6–17 mth)	9.6 ± 4.9 mm	10 (91%)
Means 2016 [39]	Prospective	Artificial conduit VS Nerve allograft; A/B	15 (A6: B9)	12 mm (5–20 mm)	12 mth	A: 5 ± 1 mm; B: 8 ± 5 mm	A:6 (100%); B:7 (78%)
Rbia 2019 [40]	Retrospective	Artificial conduit VS Nerve allograft; A/B	37 (A:19, B:18)	A:14 ± 4.9 mm; B: 18.4 ± 9.3 mm	<12 mth	A: 9.8 ± 3.8 mm B: 8.5 ± 3.7 mm	A:14 (74%); B:17 (94%)
Guo 2013 [41]	Prospective	Nerve allograft	5	23 mm (18–28 mm)	12 mth	6 ± 0.6 mm	5 (100%)
Ingari 2015 [41]	Retrospective	Nerve allograft	37	11 mm (5–15 mm)	13 days (0–215) days	7.1 ± 2.9 mm (n = 19)	31 (84%)
Rinker 2017 [42]	Retrospective	Nerve allograft	50	35 mm (25–50 mm)	11 mth	9 ± 4 mm	32 (64%)
Taras 2013 [43]	Prospective	Nerve allograft	18	11 mm (5–30 mm)	15 mth (>12 mth)	7.1 ± 1.1 mm	14 (78%)
Karabekmez 2009 [44]	Prospective	Nerve allograft	10	2.23 cm (0.5–3 cm)	9 mth (5–12 mth)	5.5 ± 2.5 mm (n = 8)	10 (100%)
He 2015 [45]	Prospective	Nerve allograft VS Neurorrhaphy; A/B	218 (A: 95 B: 123)	1.80 cm (1–5 cm)	6 mth	A: 12.81 ± 5.99 mm	A:68 (72%); B:73 (59%)
Ignazio 2010 [46]	Retrospective	Autologous vein graft	21	2.2 cm (1–3.5 cm)	>18 mth (mean, 43 mth; 18–96 mth)	8 ± 3.4 mm (n = 15)	14 (67%)
Norris 1988 [47]	Retrospective	Autologous vein graft	8	15–25 mm	11 mth	11.9 ± 4.7 mm (n = 7)	5 (63%)
Tos 2012 [48]	Retrospective	Autologous vein graft	8	1.2 cm (0.5–1.5 cm)	51 mth	-	7 (88%)
Pereira 1991 [49]	Retrospective	Autologous vein graft VS Neurorrhaphy; A/B	A: 12; B: 29	-	A: 6–40 mth; B: 8–64 mth	A:5.4 ± 2.6 mm B:11 ± 5.9 mm	A:12 (100%) B:23 (79%)
Laveaux 2011 [50]	Retrospective	Autologous vein graft	12	15 mm (3–25 mm)	50 mth (11–106 mth)	10.8 ± 2 mm (n = 11)	11 (92%)
Lee 2008 [51]	Retrospective	Autologous vein graft	3	1.43 cm (0.8–2.5 cm)	8.58 Y (2.75–12 Y)	4.7 ± 1.2 mm	2 (67%)
Risitano 2002 [52]	Retrospective	Autologous vein graft	22	1.39 cm (1–3 cm)	>12 mth	-	11 (50%)
Tang 1993 [53]	Retrospective	Autologous vein graft	18	2 cm (0.5–5.8 cm)	2 Y (18–33 mth)	5.1 ± 1.5 mm (n = 11)	11 (61%)
Walton 1989 [54]	Retrospective	Autologous vein graft	18	1.7 cm (1–3 cm)	13.6 mth (14 mth–2 Y)	4.5 ± 1.5 mm (n = 12)	12 (67%)
Manoli 2014 [55]	Retrospective	Autologous vein graft VS Neurorrhaphy VS Autologous nerve graft; A/B/C	46(A:17; B:22; C: 14)	10-60 mm	12-58mth	A:5 mm; B:3 mm; C: 5 mm	-
Chiu 1990 [56]	Prospective	Neurorrhaphy VS Autologous vein graft VS Autologous nerve graft; A/B/C	26 (A:12; B:10; C:4)	<3 cm	27.4 mth (6–72 mth)	A:7.4 ± 1.54 mm; B:11.1 ± 3.4 mm; C:9.0 ± 1 mm	A:12 (100%); B:8 (80%); C:4 (100%)
Calcagnotto 2006 [57]	Prospective	Autologous nerve graft VS Autologous vein graft; A/B	50 (A:25 B:25)	A:15.3 ± 3.8 mm B:14.6 ± 5.5 mm	10.2 ± 1.4 mth	A:8 mm (6–13 mm) B:10 mm (7–15 mm)	
Alligand-perrin 2011 [58]	Retrospective	Autologous vein graft	53	-	25.75 mth (16–39 mth)	10.3 mm (3–22 mm)	47 (89.5%)
Ederer 2023 [59]	Retrospective	Autologous vein graft	43	9-60 mm	25mth	7.0 mm	83.7%
Laveaux 2010 [60]	Retrospective	Autologous vein graft VS Autologous nerve graft; A/B	32 (A:17; B:15)	A:17 mm (5–30 mm) B:18 mm (10–30 mm)	A:62 mth B:202 mth (>11 mth)	A: 13.7 ± 4.4 mm B: 10.9 ± 5 mm	A:11 (65%); B:12 (80%)
Rose 1989 [61]	Retrospective (case report)	Autologous nerve graft	14	4.4 cm (2.5–9.0 cm)	28.3 mth (8–43 mth)	8.3 ± 3.9 mm (3–11 mm)	12(85.7%)
Li 2017 [62]	Retrospective	Autologous nerve graft	23	-	16 mth (5–32 mth)	6.8 mm (3–9 mm)	23 (100%)
Chen 2013 [63]	Retrospective	Neurorrhaphy VS Autologous nerve graft; A/B	A:21; B:31	A:2.3 cm (1.4–3.5 cm); B:2.4 cm (1.4–3.6 cm)	A:25 mth (20–26 mth); B:23 mth (19–27 mth)	A:6.4 ± 1.0 mm B: 9.2 ± 1.8 mm	A:21 (100%)
Chevrollier 2014 [64]	Retrospective	Autologous nerve graft	16	38 mm (15–60 mm)	27 mth (6–56 mth)	8.3 ± 5.8 mm (n = 12)	9 (56%)
Kim 2015 [65]	Retrospective	Autologous nerve graft	30	-	27 mth (24–37 mth)	5.9 ± 0.9 mm	30 (100%)
Mcfarlane 1976 [66]	Retrospective	Autologous nerve graft	13	2 cm (1.5–3.5 cm)	7–23 mth	14.9 ± 5.5 mm (n = 11)	4 (31%)
Nunley 1989 [67]	Retrospective	Autologous nerve graft	21	2.5 cm (1.5–4 cm)	57 mth (24–89 mth)	8.9 ± 3.6 mm (n = 18)	-
Pilanci 2014 [68]	Retrospective	Autologous nerve graft	15	1.81 cm (0.75–3 cm)	20.7 mth (9.3–41 mth)	7.1 ± 3.3 mm	15 (100%)
Bekir 2017 [69]	Retrospective	Autologous nerve graft	13	18.5 mm (15–25 mm)	35.7 mth	5.9 ± 2.2 mm	13 (100%)
Inoue 2002 [70]	Retrospective	Autologous nerve graft	3	1.3 cm (1–1.5 cm)	9.7 mth (6–12 mth)	5.3 ± 1.2 mm	3 (100%)
Young 1981 [71]	Retrospective	Autologous nerve graft	33	-	>6 mth		11 (33%)
Meek 2005 [72]	Retrospective	Autologous nerve graft	17	-	18 mth–10 Y		3 (18%)
Acar 2018 [73]	Retrospective	Neurorrhaphy	138	-	14 mth (10–20 mth)		69 (50%)
Alghazal 1994 [74]	Retrospective	Neurorrhaphy	88	-	8–32 mth		80 (91%)
Altissimi 1991 [75]	Retrospective	Neurorrhaphy	54	-	1–7 Y		40 (74%)
Efstathopoulos 1995 [74]	Retrospective	Neurorrhaphy	64	-			46 (72%)
Fakin 2016 [76]	Retrospective	Neurorrhaphy	93	-	42 mth	10.6 ± 4.5 mm	
Poppen 1979 [77]	Retrospective	Neurorrhaphy	74	-	10.9 Y (5–13.5 Y)	16.4 ± 11 mm	47 (96%)
Sladana 2017 [78]	Retrospective	Neurorrhaphy	193	-	30 mth		59 (31%)
Sullivan 1985 [79]	Retrospective	Neurorrhaphy	43	-	13 mth (6 mth–8 Y)	9.6 ± 4.3 mm (n = 33)	32 (74%)
Bulut 2016 [80]	Retrospective	Neurorrhaphy	96	-	21.4 mth (6–56 mth)		87 (91%)
Young 1993 [71]	Prospective	Neurorrhaphy	34	-	10 mth	10 mm	30 (88%)
Segalman 2001 [81]	Retrospective	Neurorrhaphy	19	-	>1 Y	5.5 ± 2.3 mm (n = 10)	11 (58%)
Vahvanen 1981 [82]	Retrospective	Neurorrhaphy	18	-	7.5 Y (2–18 y)		18 (100%)
Wang 1996 [83]	Retrospective	Neurorrhaphy VS Autologous nerve graft; A/B	90 (A:76; B:14)	1.5–6 cm	>1 Y	A:6 ± 3.7 mm (n = 29); B:7 ± 7.4 mm (n = 5)	A:64 (84%); B:9 (64%)
Mennen 1998 [84]	Retrospective	Neurorrhaphy	5	-	>3 mth		4 (80%)
Voche 2005 [85]	Retrospective	Neurorrhaphy	11	2.5 cm (1.5 –4 cm)	>9 mth	9.1 ± 1.6 m	11 (100%)
Landwehrs 2008 [86]	Retrospective	Neurorrhaphy	5	-	21 mth (11–39 mth)	6 ± 0 mm (n = 2)	
Artiaco 2010 [87]	Retrospective	Neurorrhaphy	7	-	35 mth (8–60 mth)	12.7 ± 3.3 mm	6 (86%)
Chow 1993 [88]	Prospective	Neurorrhaphy	108	-	3, 6, 12, 18, 24 mth		65 (90%)

To map the evolving research landscape of NGCs, we conducted a bibliometric analysis of literature published over the past five years in the Web of Science database. Keyword analysis revealed strong research focus on “functional regeneration,” “biocompatibility,” “biomaterials,” “mechanical property,” and “3D printing.” Furthermore, keyword clustering identified “commercializable nerve guidance conduit” as a prominent research direction, which reinforces the urgent clinical need we identified in our prior meta-analysis for novel solutions that surpass existing technological limitations. In this context, metal ion-based strategies emerge as a highly promising approach. Their defined chemical properties, excellent biocompatibility, and compatibility with advanced manufacturing position metal ions as key enablers for developing next-generation, high-performance, and clinically translatable NGCs.

Guided by these insights, the following sections provide a detailed analysis of metal ions in PNR, examining their roles in development, concentration-dependent effects, and molecular mechanisms. This foundation further supports a discussion on translating these principles into therapeutic applications through advanced manufacturing technologies.

## 3 Spatiotemporal regulation of peripheral nerve development through metal ions

**Fig. 2:**
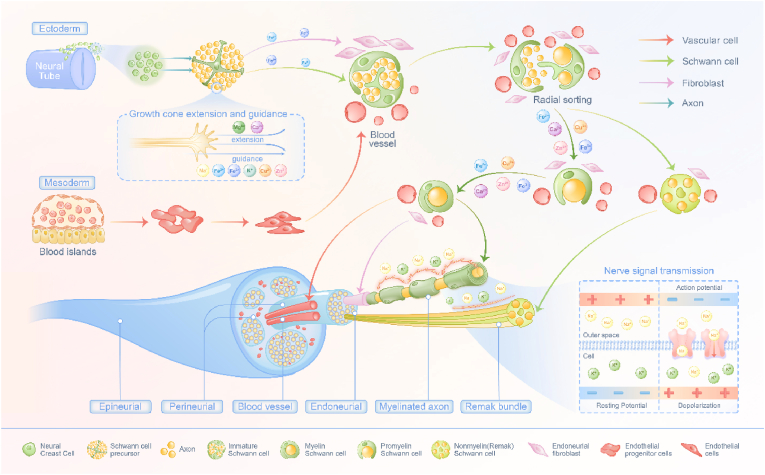
Involvement of metal ions in peripheral nerve development.

PNS derives primarily from the neural crest, a transient embryonic structure renowned for its developmental plasticity and extensive contributions [89]. Neural crest cells migrate and differentiate into key PNS components, including sensory neurons, autonomic ganglia, Schwann cells, and endoneurial fibroblasts. The connective tissue sheaths of peripheral nerves, such as the perineurium and epineurium, derive mainly from mesoderm-derived mesenchymal cells, while the associated vasculature arises from mesodermal blood islands [90,91]. This section systematically reviews the development of these cellular subpopulations and the regulatory influences of metal ions throughout this process (Fig. 2).

### 3.1 Axons: the pioneers of neural pathfinding

Axonal pathfinding is directed by growth cones that integrate guidance cues, including Netrin, Slit, Semaphorins, and Ephrin [92,93]. Metal ions, particularly calcium, are now recognized as central players in axonal guidance, operating within the framework of molecular guidance. Calcium ions are central to this guidance, with local concentration gradients dictating turning behaviors (attraction at 200 nM, repulsion at 75 nM) via cytoskeletal rearrangements and membrane trafficking [[94], [95], [96], [97]]. Initially, moderate calcium signals create a permissive state by inhibiting RhoA via the CaN/PP1 axis, thus preventing excessive contractility [98,99]. Subsequently, confined high-calcium microdomains initiate polarized extension through CaMKII-mediated Rac/Cdc42 activation, leading to localized cytoskeletal stabilization [100,101]. In parallel, calcium gradients orchestrate vesicle cycling, stimulating exocytic insertion at the leading edge while synchronizing endocytic removal from the sides and rear [102,103]. Other metal ions exert indirect influences: sodium and potassium channels regulate membrane potential to shape growth cone behavior [104,105]; iron supports ATP production to meet biosynthetic demands [18,106]; magnesium and zinc contribute to synaptic refinement and precise guidance, respectively [16,17].

### 3.2 Schwann cells: myelination architects and axonal chaperones

Schwann cell development begins with neural crest-derived Schwann cell precursors (SCPs), which depend on axonal Neuregulin 1 (NRG1) for survival before differentiating into immature Schwann cells (iSCs) [[107], [108], [109]]. The fate of iSCs is determined by axon caliber and NRG1-type III signaling, leading to either myelinating or non-myelinating (Remak) phenotypes [10,110]. Mature Schwann cells maintain their survival through autocrine signaling, laminin interactions, and persistent axonal support, and they contribute to nerve structure and metabolic homeostasis [[111], [112], [113]]. Iron and calcium signaling act synergistically to regulate Schwann cell development and myelination. Iron supports bioenergetics and lipid synthesis, activates the cAMP/CREB pathway for myelin protein expression, and induces protective oxidative responses [114]. Calcium signaling, triggered by axonal ATP via P2RY2, enhances mitochondrial metabolism to cooperate with iron-dependent processes in myelination [115].

### 3.3 Fibroblasts: maintaining neural microenvironment integrity

The connective tissue sheath of peripheral nerves comprises endoneurial, perineurial, and epineurial fibroblasts, each with distinct origins and functions [116]. Endoneurial fibroblasts (EFs) derive from the neural crest and perform roles in collagen synthesis and immune modulation [91]. Perineurial fibroblasts (PFs), of mesenchymal origin, form the blood-nerve barrier [[117], [118], [119]], while epineurial fibroblasts (EPFs) construct the protective collagenous scaffold [120,121]. This ontogenetic hierarchy dictates specialization in barrier function and microenvironment maintenance.

Current understanding of fibroblast development in the peripheral nerve remains limited, a challenge compounded by their diverse cellular origins. Direct evidence linking metal ions to the development of specific fibroblast subpopulations in this context is currently lacking. To address this gap, we draw on studies of metal ion effects on progenitor cells of various lineages, allowing us to infer their potential roles in fibroblast development, differentiation, and function. A subset of fibroblasts in peripheral nerves originates from mesenchymal stem cells (MSCs). Metal ions have been demonstrated to directly regulate critical functions of MSCs, including cell adhesion, migration, and extracellular matrix (ECM) remodeling. Although direct evidence that metal ions drive MSC differentiation into fibroblasts is still absent, these regulatory mechanisms offer valuable insight. Physiologically, magnesium and calcium ions bind integrin sites to modulate ECM interactions [122]. while zinc acts as an essential cofactor for matrix metalloproteinases (MMPs), directly controlling ECM proteolysis and thereby influencing cell migration and tissue remodeling [123]. Another, smaller fibroblast population derives from neural crest cells. These cells can be directed toward glial or myofibroblast lineages by factors such as Bmp4, Nrg1, and Delta-Fc [124]. Metal ions may directly or indirectly regulate the expression or activity of these key factors, thus influencing cell fate decisions [125,126].

### 3.4 Vascular endothelial cells: companions in axonal pathfinding

Vascular endothelial cells arise from mesodermal blood islands. During development, an initial neurovascular plexus forms alongside the differentiation of SCPs into iSCs, guided by iSC-derived paracrine signals [108,127]. Blood vessels maintain a close association with growing axons, sharing guidance cues such as ephrins and semaphorins, and vascular-derived VEGF promotes axonal maturation [[128], [129], [130]].

The development of the PNS is orchestrated by dynamic calcium ion gradients, which guide axon pathfinding through rhythmic fluctuations. This key regulatory system functions not only on its own but also within a network of essential cations like magnesium, zinc, iron, and copper. Together, they guarantee accurate neural development and maturation.

## 4 Visualizing the current landscape of metal ions in peripheral nerve research via bibliometric analysis

While the critical roles of metal ions in peripheral nerve development are well-documented, the existing knowledge is dispersed across disparate studies. To integrate these insights and establish a coherent framework, we conducted a bibliometric analysis to map the research landscape, pinpoint key foci, and identify outstanding challenges.

Utilizing the Web of Science Core Collection (WoSCC), we conducted a comprehensive search using keywords: “peripheral nerve regeneration,” “peripheral nerve repair,” “neural tissue engineering,” “nerve conduit,” and “metal ions.” The search timeframe was restricted from January 1, 2005, to August 8, 2025, and document types were limited to articles and reviews. After deduplication, we compiled a final set of 238 articles for analysis.

**Fig. 3:**
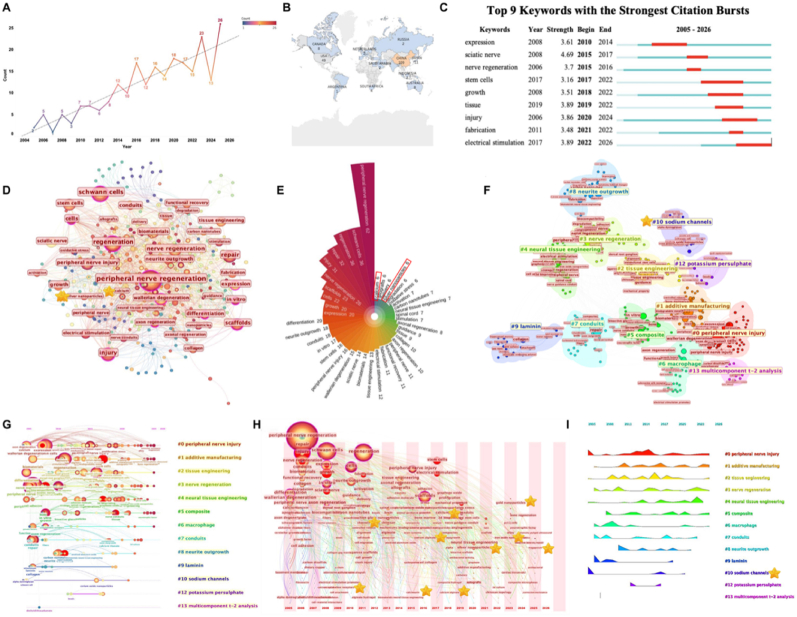
**Growing research interest in metal ion studies for peripheral nerve regeneration.** (A) Annual publication counts of metal ion-involved peripheral nerve regeneration research. (B) Relative publication output by country/region. (C) Burst strength and time span of the top 9 keywords. (D) Occurrence frequency of keywords across the literatures. (E) Top 40 keywords in related researches. (F) Keyword Clustering Analysis. (G-I) Temporal distribution of keywords.

Quantifying the annual number of publications revealed a consistent growth in research activities involving metal ions for peripheral nerve applications (Fig. 3A). Notably, China (109 articles) and the United States (49 articles) emerged as the primary contributors (Fig. 3B). Within the 238 articles, we observed keywords exhibiting citation bursts during different time periods. In recent years, “electrical stimulation” appeared as a potential hot topic related to metal ions (Fig. 3C). Visualization of keywords with an occurrence frequency ≥5 showed that “peripheral nerve regeneration” is the core focus (Fig. 3D–Table 2). The most common keywords included “peripheral nerve regeneration,” “Schwann cells,” “repair,” “scaffold,” “differentiation,” “neurite outgrowth,” “conduit,” “stem cells,” etc. (Fig. 3E). Keyword clustering formed 14 distinct clusters, illustrating a diverse evolution from fundamental metal ion research towards integrated tissue engineering approaches (Fig. 3F–I). Historically, research in neural regeneration prioritized developmentally pivotal ions, such as calcium, and fundamental mechanisms like Wallerian degeneration. More recent investigations, however, have shifted focus toward novel metal ions and their emerging applications in advanced tissue engineering strategies.

**Table 2:** Top100 keywords based on bibliometric analysis.

Keyword	Frequence	Year
**peripheral nerve regeneration**	62	2006
**regeneration**	36	2011
**schwann cells**	36	2008
**repair**	32	2006
**injury**	31	2006
**nerve regeneration**	26	2006
**scaffolds**	23	2016
**cells**	22	2010
**differentiation**	20	2005
**expression**	20	2008
**growth**	20	2008
**conduits**	18	2006
**neurite outgrowth**	18	2010
**in vitro**	17	2008
**peripheral nerve injury**	16	2015
**stem cells**	16	2017
**wallerian degeneration**	15	2005
**biomaterials**	14	2006
**sciatic nerve**	14	2008
**tissue engineering**	13	2014
**electrical stimulation**	12	2017
**peripheral nerve**	11	2005
**functional recovery**	11	2006
**fabrication**	11	2011
**collagen**	10	2006
**axon regeneration**	10	2008
**guidance**	9	2010
**tissue**	9	2019
**axonal regeneration**	8	2014
**activation**	7	2011
**carbon nanotubes**	7	2010
**neural tissue engineering**	7	2020
**spinal cord**	7	2013
**stimulation**	7	2010
**calcium**	6	2005
**allografts**	6	2014
**delivery**	6	2011
**silver nanoparticles**	6	2020
**degradation**	6	2006
**oxidative stress**	6	2019
**nerve conduits**	6	2019
**strategy**	6	2011
**nanoparticles**	6	2013
**brain**	6	2013
**biocompatibility**	5	2008
**bone**	5	2008
**chitosan**	5	2013
**adhesion**	5	2016
**mesenchymal stem cells**	5	2019
**neurons**	5	2014
**growth factor**	5	2006
**in vivo**	5	2016
**neuronal differentiation**	5	2021
**bioactive glass**	4	2011
**apoptosis**	4	2005
**axon degeneration**	4	2005
**proliferation**	4	2018
**alginate**	4	2013
**graphene oxide**	4	2018
**cerium oxide nanoparticles**	4	2016
**system**	4	2016
**peripheral nerve repair**	4	2015
**mechanisms**	4	2013
**nerve guidance conduit**	4	2019
**mechanical property**	4	2018
**release**	4	2018
**neural differentiation**	4	2021
**cell adhesion**	3	2006
**calcium titanate**	3	2018
**3d bioprinting**	3	2018
**laminin**	3	2006
**bone marrow**	3	2016
**angiogenesis**	3	2019
**channels**	3	2011
**alignment**	3	2011
**fibers**	3	2008
**controlled release**	3	2011
**schwann cell**	3	2005
**cell death**	3	2011
**behavior**	3	2017
**nerve guidance conduits**	3	2025
**agarose scaffolds**	2	2011
**dorsal root ganglion**	2	2009
**gold nanoparticles**	2	2023
**bone regeneration**	2	2024
**composites**	2	2018
**additive manufacturing**	2	2020
**crush injury**	2	2011
**aligned collagen**	2	2016
**alloy**	2	2023
**autografts**	2	2020
**attachment**	2	2006
**cell growth**	2	2011
**adhesion molecule**	2	2014
**biodegradable metal**	2	2015
**axonal guidance growth**	2	2015
**axon outgrowth**	2	2018
**alpha**	2	2018
**beads**	2	2012
**pc12 cells**	2	2023

Beyond this macroscopic perspective, the following section elucidates the specific mechanisms by which metal ions promote PNR.

## 5 Spatiotemporal regulation of peripheral nerve regeneration via metal ions

**Fig. 4:**
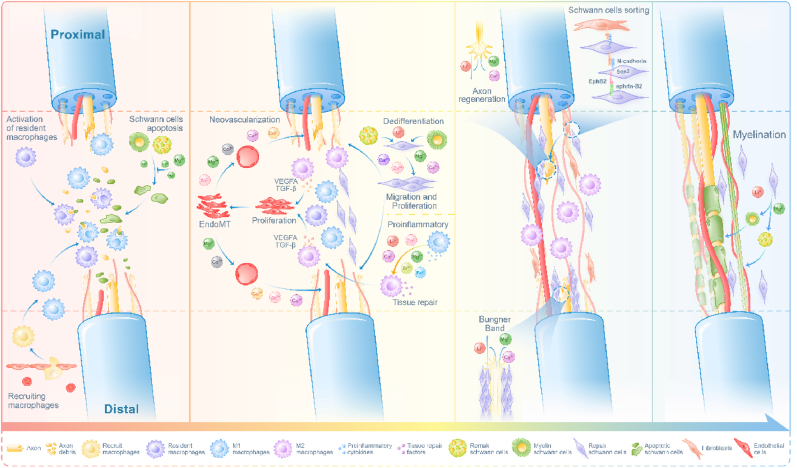
Involvement of metal ions in peripheral nerve regeneration.

Following PNI, the PNS activates a coordinated repair process involving axons, Schwann cells, immune cells, fibroblasts, and endothelial cells [131]. The early phase features Wallerian degeneration, which activates resident macrophages and recruits monocytes to the site [132]. A critical transition then occurs as macrophages shift from a pro-inflammatory (M1) to a pro-repair (M2) phenotype, secreting factors like VEGF-A and TGF-β to promote angiogenesis [133,134]. Concurrently, Schwann cells dedifferentiate into repair subtypes [135,136]. During regeneration, these cells form aligned cords (guided by fibroblasts) and Büngner bands to direct axonal regrowth along new vasculature [137]. In the final maturation phase, axons reconnect with their targets, and Schwann cells redifferentiate into mature phenotypes, ultimately restoring nerve structure and function (Fig. 4).

### 5.1 Schwann cells: orchestrators of regenerative responses

The regenerative capacity of PNS, which contrasts markedly with that of the central nervous system, derives largely from the exceptional plasticity of Schwann cells. This plasticity enables their pivotal transition into a reparative phenotype after injury. The cells play a multifaceted role: they orchestrate immune cell recruitment, interact with fibroblasts to form guidance structures, and secrete neurotrophic factors that directly promote axonal elongation [138,139]. After PNI, Schwann cells dedifferentiate into two main states: proliferating Schwann cells (pSCs) that repopulate the denervated area, and repair Schwann cells (rSCs) that direct regeneration. rSCs initiate repair by secreting cytokines to recruit immune cells and clear myelin debris through biphasic mechanisms—first via actin-based segmentation and autophagy, then via TAM receptor-mediated phagocytosis with macrophages [136,140,141]. Simultaneously, rSCs provide neurotrophic support and differentiate into specialized subtypes [142]. Guided by TGFβ signaling and fibroblast-derived Ephrin-B, bridge Schwann cells (bSCs) form regenerative cords to guide axons, while distal Schwann cells (dSCs) assemble Büngner bands as growth scaffolds. Terminal Schwann cells (tSCs) aid final reinnervation. Although rSCs eventually redifferentiate upon regeneration, their reparative function is time-limited [143]. A key challenge is to effectively drive this process, maximize axonal growth within the critical window, and precisely control redifferentiation to restore structure and function.

Notably, metal ions have gained recognition as master regulators that can orchestrate this complex regenerative sequence, providing a precision toolset to address existing challenges. A pivotal initial step involves directing Schwann cell fate towards a repair phenotype, where suppressing apoptosis and enabling their phenotypic transition is fundamental to initiating the proliferative and migratory programs necessary for regeneration.

Magnesium, at low concentrations, facilitates Schwann cell-mediated repair by suppressing apoptosis through the upregulation of Bcl-2/Bcl-xL and inhibition of caspase-3 activity, thereby enhancing cell migration and proliferation in the early phase of PNI [144,145].

Lithium orchestrates Schwann cell plasticity via dual inhibition of GSK-3β, thereby coordinating both dedifferentiation and redifferentiation programs. This dual inhibition is achieved through direct competition with magnesium at the kinase's catalytic site and indirectly via Akt-mediated Ser-9 phosphorylation, which collectively amplify Wnt/β-catenin signaling [[146], [147], [148]]. Consequently, lithium promotes dedifferentiation by upregulating c-Jun [149,150], while simultaneously facilitating redifferentiation through the activation of myelination-related genes [151]. This bifurcated regulatory capacity allows lithium to critically modulate Schwann cell fate transitions during PNR.

Iron exerts a concentration-dependent duality on Schwann cell fate. At low concentrations, it elicits a pro-oxidative signal that elevates intracellular cAMP, activating the PKA-CREB axis to upregulate myelin proteins. Conversely, high iron concentrations increase expression of the dedifferentiation marker p75NTR, which, via interaction with phosphodiesterase 4, reduces cAMP levels and promotes a repair phenotype [114].

Recognizing the insufficient efficacy of single metal ions in directing Schwann cell plasticity, recent research has pivoted to leveraging the synergistic effects of composite metal ions. Zinc-gallium composite ions significantly enhance migration efficiency by remodeling the cytoskeleton (Rho GTPases) of the Schwann cells, paving the way for axonal extension [152]. Lithium-magnesium-silicon composite ions activate the β-catenin signaling pathway, driving the expression of myelin core proteins (PMP22, MBP) and neurotrophic factors (NGF), while inhibiting immature markers such as NCAM, promoting the transformation of Schwann cells into a myelinating phenotype [153]. Zinc-silicon and calcium-magnesium-silicon composite systems further enhance the axonal regeneration microenvironment through S100 upregulation-mediated Schwann cell differentiation into myelinating phenotypes, which subsequently triggers BDNF secretion to guide efficient axonal regeneration [154].

### 5.2 Fibroblasts: dynamic regulators of neural microenvironment homeostasis

As established in developmental studies, EFs, PFs, and ENFs exhibit distinct embryonic origins and differentiation pathways, resulting in specialized functional roles in mature nerves. During the early regenerative phase, PFs rapidly migrate to the injury site through Ephrin-B2/EphB2 signaling, guiding rSCs to assemble into aligned cord-like structures that initiate axonal regrowth [118,155]. ENFs exhibit immune surveillance functions and cooperate with rSCs to maintain regenerating axons within Büngner bands during their distal extension [156,157]. EFs primarily secrete extracellular matrix components that provide a physical scaffold for axonal regrowth [158]. However, under pathological conditions such as chronic inflammation, repeated mechanical injury, or metabolic abnormalities, these cells abnormally transform into contractile myofibroblasts, leading to collagen ratio imbalance and excessive secretion of inhibitory molecules [159]. This results in the formation of a high-stiffness fibrotic scar, which not only physically obstructs axon extension but also induces growth cone collapse, ultimately causing regeneration failure [160].

The strategic application of metal ions to modulate fibroblast polarity represents a promising direction for functional nerve regeneration. By engaging fibroblasts early to guide Schwann cell migration and angiogenesis, and later to reconstruct the connective tissue sheaths (endoneurium, perineurium, epineurium) for barrier restoration, metal ions can potentially reshape the neural microenvironment. Although their specific effects on nerve-derived fibroblasts remain unexplored, insights from other tissues provide valuable references. For instance, magnesium enhances fibroblast motility and adhesion by upregulating ITGB1 to activate FAK and downstream pathways [161,162], and by modulating Erk2 within the MAPK cascade [163]. Conversely, gadolinium promotes fibroblast proliferation and myofibroblast differentiation, driving fibrosis and highlighting its pathways as potential anti-scarring targets [[164], [165], [166], [167]].

### 5.3 Immune cells: modulators of the neural microenvironment

Following PNI, a precisely timed immune response unfolds. The initial 24-48 h are marked by Wallerian degeneration, microvascular disruption, and acute inflammation, establishing a hypoxic milieu that primes the microenvironment [168]. This pro-inflammatory phase (days 0-7) is dominated by infiltrating neutrophils, recruited via the CXCL1/CXCL2-CXCR2 axis, and M1-type macrophages that peak by day 7. These macrophages secrete TNF-α and IL-1β, collaborating with rSCs to clear debris [169]. Subsequently, a reparative program supersedes this acute response over the following 1-2 weeks [170,171]. Persistent hypoxia drives macrophage polarization toward an M2 phenotype, which promotes angiogenesis via the HIF-1α/VEGF axis and secretes TGF-β and BDNF to guide rSCs and axons while suppressing inflammation [168]. In later stages, M2 macrophages dominate tissue reconstruction by activating MMPs to restrain fibroblast activation, promoting orderly ECM remodeling, and regulating remyelination [172]. The adaptive immune system also contributes during this phase, with T cells mediating cellular communication and B cells participating in humoral immunity [173].

The precise timing of the M1-to-M2 macrophage transition is critical for PNR, balancing debris clearance with repair. Magnesium ions orchestrate this anti-inflammatory shift via a defined signaling cascade: entry through the TRPM7 channel activates the PI3K-AKT1 pathway. This signaling node concurrently dampens NF-κB activity via TLR4-MyD88 downregulation, selectively curbs pro-inflammatory MAPK signaling (p38/JNK), and scavenges LPS-induced ROS, collectively resolving inflammation and facilitating tissue repair [174,175].

Iron ions exert concentration-dependent, multi-layered control over macrophage polarization. At low concentrations, they suppress M1 polarization by inhibiting IκBα degradation and subsequent NF-κB p65 nuclear translocation [176]. Intermediate concentrations achieve selective inhibition of M1 commitment by blocking STAT1 signaling while preserving STAT6 activity [177]. Conversely, high iron levels promote a robust pro-inflammatory phenotype through synergistic mechanisms involving MAPK activation, hepcidin-mediated STAT6 suppression, IRF3 upregulation, and enhanced ROS signaling [178,179].

Beyond the well-characterized roles of magnesium and iron, several other metal ions significantly modulate macrophage polarization via distinct pathways. Strontium ions, released from bioactive glasses, enhance mitochondrial function to boost oxidative metabolism, thereby upregulating M2 markers (IL-10, CD206) and suppressing M1 markers (TNF-α, iNOS) [180]. Zinc ions concurrently inhibit the TLR/MyD88 pathway to curb M1 polarization and activate STAT6 to promote M2 gene expression [181]. Leveraging the potent effects of magnesium, composite ion systems have been developed. For instance, lithium-magnesium-silicon composites increase the M2/M1 ratio (evidenced by CD206/Arg-1 upregulation and iNOS/TNF-α downregulation), accelerating nerve regeneration and motor function recovery [153]. Similarly, calcium-magnesium-silicon synergy potently upregulates M2 genes while inhibiting M1 factors, dynamically balancing inflammation and repair [182].

While the effect of metal ions on macrophage M1/M2 polarization is a focus in nerve regeneration, their influence on the myriad of other involved immune cells constitutes an open and compelling field for future research.

### 5.4 Endothelial cells: paving the path for regeneration

In the three-dimensional regenerative landscape, Schwann cells depend on guidance from cellular partners, particularly endothelial cells (ECs), which act as critical pathfinding beacons [183]. A hypoxic microenvironment, instigated by pro-inflammatory macrophages, activates the HIF-1α signaling axis to coordinately release VEGF and TGF-β [133]. VEGF directs angiogenesis by recruiting migratory tip cells, while TGF-β promotes endothelial-to-mesenchymal transition (EndoMT) to enhance their mobility [184]. Following MMP-mediated ECM degradation by tip cells, stalk cells proliferate and form patent lumens, which are subsequently stabilized by recruited pericytes [185]. Furthermore, endothelial cell-derived exosomes (EC-EXO) enhance the Schwann cell repair phenotype via miR-199-5p and PI3K/AKT signaling [186]. This orchestrated cascade ultimately enables repair Schwann cells to migrate along the nascent vascular scaffold, bridging the injury site and guiding axonal regeneration.

Metal ions orchestrate angiogenesis through sophisticated regulatory networks, primarily via direct endothelial interactions and indirect macrophage-mediated pathways. Zinc promotes angiogenesis by directly upregulating VEGF and HIF-1α in endothelial cells while concurrently polarizing macrophages toward a VEGF-secreting M2 phenotype [181,187]. Copper employs a multifaceted strategy involving VEGF upregulation [178,184], angiogenin modulation [188,189], matrix metalloproteinase activation [130], and direct stimulation of endothelial migration and proliferation [12]. Both ions converge on the VEGF signaling cascade, modulating key pathways such as Ras/MAPK, FAK/paxillin, PI3K/AKT, and PLCγ to coordinately regulate proliferation, migration, survival, and permeability [190]. Additionally, cobalt enhances angiogenesis via a tripartite mechanism of hypoxia mimicry, immunomodulation, and direct endothelial activation, while lithium acts through Wnt/β-catenin pathway stimulation [191,192]. Collectively, these distinct yet interconnected mechanisms establish a finely balanced, multi-scale regulatory network for angiogenesis.

### 5.5 Axons: the cornerstone of regeneration

After PNI, a pioneering growth cone emerges from the swollen axon stump. This dynamic structure, enriched with actin filaments and microtubules, senses and responds to environmental guidance cues [193]. The rSCs and surrounding tissues secrete guidance molecules that orchestrate the growth cone's pathfinding [194]. Its advancement, driven by actin polymerization and microtubule dynamics, culminates in target reinnervation and the restoration of neural circuitry via synaptic remodeling [195].

Lithium ions promote axonal extension primarily by inhibiting GSK3β, which stabilizes β-catenin to activate regeneration genes via TCF/LEF and suppresses growth cone collapse via Smad1 interaction [192,196]. This effect is amplified by a positive feedback loop wherein lithium also activates the PI3K/Akt pathway to further inhibit GSK3β [153]. Magnesium ions enhance growth cone dynamics by activating PPAR-γ to indirectly inhibit the RhoA/ROCK pathway, thereby stabilizing microtubules and promoting extension [197]. Similar to development, calcium ions, in turn, orchestrate axonal growth through two major cascades: the Ca^2+^/CaM-AC-cAMP-PKA axis drives CREB-mediated BDNF expression to support growth cone migration [198], while CaMKII activation phosphorylates and stabilizes MAP2 to reorganize the axonal cytoskeleton [199]. Given this central role, regulating intracellular calcium ion concentration emerges as a strategic avenue for enhancing axonal regeneration.

**Fig. 5:**
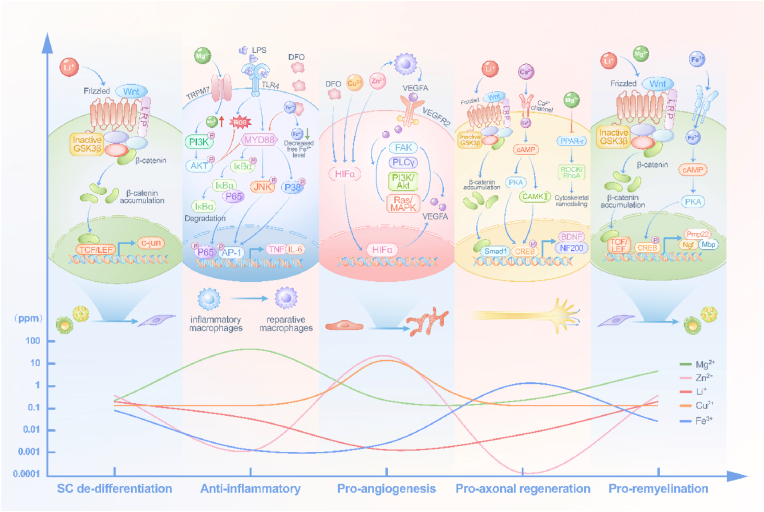
Regulatory mechanisms of metal ions in peripheral nerve regeneration.

PNR constitutes a highly orchestrated spatiotemporal process where precisely coordinated cellular and molecular events unfold across distinct yet overlapping phases. The concentration-dependent, biphasic nature of metal ions necessitates thorough investigation into their dose-specific mechanisms to fully exploit their therapeutic potential. As illustrated in Fig. 5, six key metal ions (lithium, copper, zinc, magnesium, calcium, and iron) coordinate PNR through evolutionarily conserved developmental signaling pathways, with their precise spatial and temporal regulation proving essential for successful regeneration outcomes.

During the initial phase of Schwann cell dedifferentiation, lithium ions activate the Wnt/β-catenin pathway to facilitate the transition of mature Schwann cells to a repair-prone state. The subsequent pro-inflammatory phase demonstrates how iron and magnesium fine-tune inflammatory responses through PI3K/Akt and NF-κB pathways, establishing a balanced microenvironment conducive to repair processes. As regeneration advances to angiogenesis and axonal regrowth, magnesium, lithium, zinc and copper exhibit particularly significant spatiotemporal functions. Zinc and copper support neovascularization through HIF-1α stabilization, while magnesium and lithium activate both the Wnt/β-catenin pathway and the ROCK/Rhoa pathway to guide growth cone navigation. The final remyelination phase involves iron promoting myelin protein expression via cAMP activation, complemented by lithium and magnesium supporting Schwann cell differentiation through GSK-3β inhibition.

The temporal hierarchy of signaling pathway activation ensures proper sequencing of regenerative events. The Wnt/β-catenin pathway predominates during early dedifferentiation, followed by peak activity of PI3K/Akt and NF-κB pathways during the pro-inflammatory phase. HIF-1α signaling dominates vascular regeneration, while the Wnt/β-catenin pathway reactivates during the final regenerative phase. Metal ions serve as master regulators of this sophisticated temporal sequence.

Spatial distribution patterns further refine the regulatory precision of these metal ions. Calcium ions, primarily modulated through electrical and topographical cues due to their dynamic flux, work synergistically with other metal ions to create microenvironments optimized for each regenerative phase. This comprehensive review establishes specific concentration ranges for lithium, copper, zinc, magnesium, and iron (Table 3) to inform targeted therapeutic approaches that respect the spatiotemporal complexity of nerve regeneration.

**Table 3:** The concentration ranges of metal ions in regulating peripheral nerve-related cells.

	Function	magnesium	zinc	iron	lithium	copper
**Axons**	Pro-axonal regeneration	0.06-0.24 ppm [200]	0.000065-0.0026 [201]	1.4 ppm [202]	0.0014-0.007 ppm [203]	/
**Schwann cells**	SC de-differentiation	2-5 ppm [144]	0.4 ppm [204,205]	0.084 ppm [114]	<0.21 ppm [149]	/
	Pro-remyelination	/	/	0.028 ppm [114]	0.07 ppm [151]	/
**Macrophages**	Anti-inflammatory	50-200 ppm [171,174]	0.000325-0.0013 ppm [206]	0.0014 ppm [176]	/	>0.00064 ppm [206]
**Endothelial cells**	Pro-angiogenesis	0.048-0.24 ppm [207]	25-30 ppm [208]	0.0028 ppm [209]	0.0014-0.007 ppm [210]	21- 36 ppm [211]

Through this multilayered regulation, metal ions offer a foundation for designing phase-specific therapeutic strategies that may substantially improve outcomes in peripheral nerve regeneration.

### 5.6 Single-cell analysis elucidates conserved metal ion regulation in peripheral nerve development and regeneration

Calcium, lithium, magnesium, zinc, iron, and copper are pinpointed in our systematic review as six pivotal metal ions that act as crucial regulators throughout peripheral nerve development and regeneration. However, current research, largely confined to whole-tissue level analyses, has yet to precisely delineate how these ions target specific cellular subpopulations to enact their functions during peripheral nerve development and regeneration. The advent of single-cell sequencing technologies offers a transformative opportunity to overcome this limitation [212,213]. Integrating such high-resolution data can provide an unprecedented perspective on the cellular dynamics and functional specialization governing PNR [214].

To systematically investigate the dynamic changes of six key metal ions during peripheral nerve development and injury regeneration, we integrated single-cell RNA-seq data spanning mouse developmental stages and multiple injury time points—including neonatal and adult phases, crush injuries at 1/3/7 days and transection injuries at 3/9 days [116,215,216], as well as rat acute (0/1/3/7 days) and chronic crush injuries (0/3/12/60 days) (Supplementary Tables 1 and 2) [136,217].

**Fig. 6:**
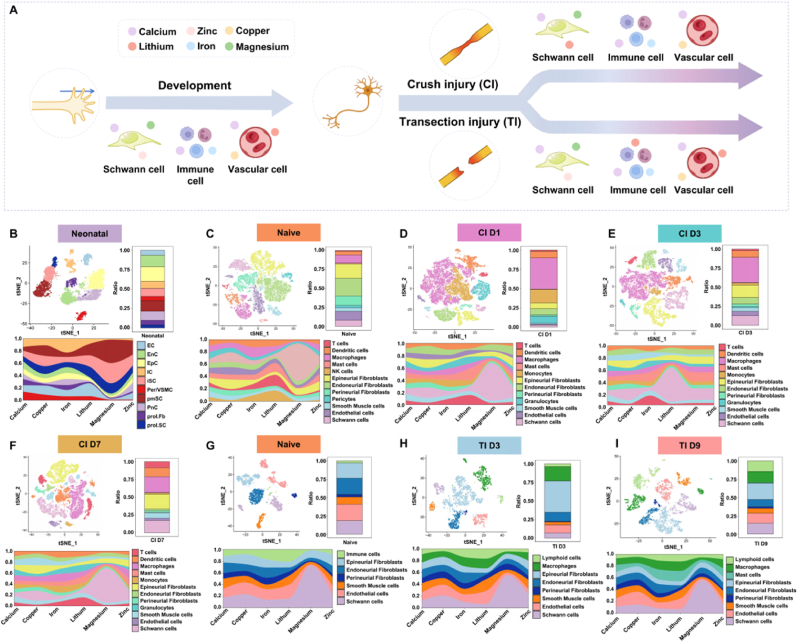
**Single-cell analysis in mice.** (A) Regulatory roles of metal ions in murine peripheral nerve development and regeneration. (B) Functional scores of metal ions in critical cellular subpopulations of neonatal group. (C-F) Functional scores of metal ions in critical cellular subpopulations of naive group and nerve crush injury group. (G-I) Functional scores of metal ions in critical cellular subpopulations of naive group and nerve transection injury group.

Integrated single-cell analyses reveal conserved functional roles of metal ions during peripheral nerve development and regeneration following crush or transection injuries (Fig. 6A, Supplementary Fig. 2). Profiling of the neonatal mouse nerve identified a complex microenvironment, which included Schwann cell subtypes (immature [iSC], premyelinating [pmSC], and proliferating [prol.SC]), epineurial, perineurial, and endoneurial fibroblasts, together with associated immune and vascular components (Fig. 6B). AUCell algorithm analysis demonstrated cell-type-specific metal ion enrichment: magnesium and zinc were significantly enriched in Schwann cells, particularly myelin-related subsets; iron was primarily associated with immune cells; copper and lithium favored vascular cells; and calcium displayed a pan-cellular distribution. During maturation to adulthood, dynamic shifts in cellular composition were observed, with fibroblasts becoming the dominant population alongside a relative reduction in Schwann cells and an increase in immune cells, likely reflecting the establishment of microenvironmental homeostasis (Fig. 6C).

The response to PNI revealed a striking conservation in metal ion distribution that transcends developmental and regenerative contexts. Despite profound cellular remodeling, including immune expansion and biphasic Schwann cell dynamics, magnesium, copper, and iron maintained a precise cellular tropism for Schwann, vascular, and immune cells, respectively. A key temporal switch was observed at day 3 post-crush, as Schwann cells transitioned from magnesium/zinc to lithium preference (Fig. 6D–F). This signature was conserved even in the slower-regenerating transection model, where delayed cellular dynamics nonetheless adhered to the same ionic patterning (Fig. 6G–I). The persistent, stable expression of calcium across all stages suggests its role as a universal regulatory signal.

**Fig. 7:**
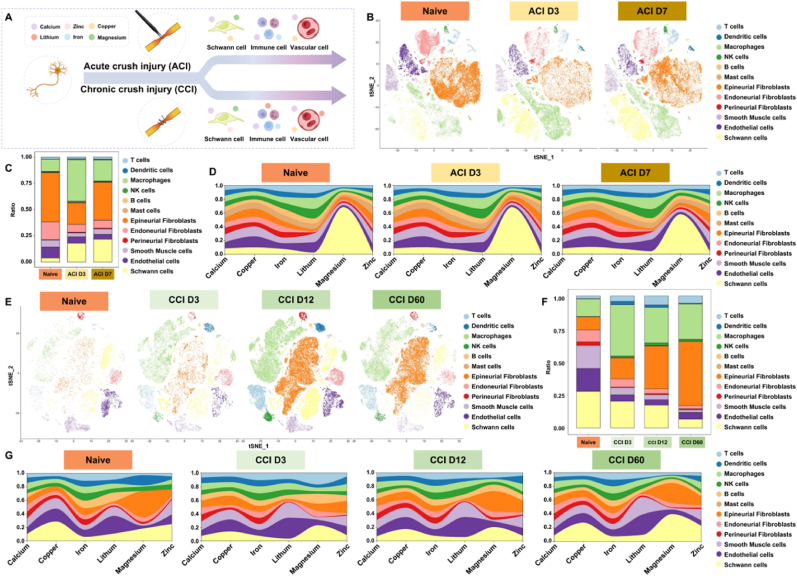
**Single-cell analysis in rat.** (A) Regulatory roles of metal ions in rat peripheral nerve development and regeneration. (B-D) Functional scores of metal ions in critical cellular subpopulations of naive group and acute crush injury group. (E-G) Functional scores of metal ions in critical cellular subpopulations of naive group and chronic crush injury group.

The functional conservation of metal ions extends beyond developmental-regenerative parallels to exhibit cross-species preservation (Fig. 7A, Supplementary Fig. 3). Single-cell profiling reveals distinct cellular dynamics in acute (ACI) versus chronic crush injury (CCI) models (Fig. 7B, C, E, F). ACI elicits rapid immune infiltration and Schwann cell proliferation. In contrast, CCI is characterized by persistent immune activation, significant epineurial fibroblast accumulation, and a reduction in Schwann cells, which collectively indicate a fibrogenic microenvironment. Notably, despite these divergent cellular landscapes, metal ions maintain evolutionarily conserved regulatory roles (Fig. 7D–G). Magnesium remains robustly associated with Schwann cell dynamics and additionally correlates with epineurial fibroblast proliferation in chronic injury. Copper and lithium predominantly modulate vascular activation and angiogenesis, while iron and lithium influence immune-mediated inflammation. Notably, the specific association of magnesium with epineurial fibroblast accumulation in CCI suggests its potential regulatory involvement in fibrogenesis (Fig. 7G). Although the mechanisms of nerve fibrosis remain elusive, this finding provides a pivotal clue for investigating magnesium's direct role in this process and establishes a framework for understanding the regeneration-fibrosis balance in chronic nerve injury.

The single-cell resolution-based functional scoring algorithm pinpointed metal ion-sensitive cellular subpopulations, thereby establishing a framework for developing spatiotemporally targeted metal ion-based therapeutic strategies.

### 5.7 Developmental cues for nerve regeneration: guiding next-generation scaffold design

**Fig. 8:**
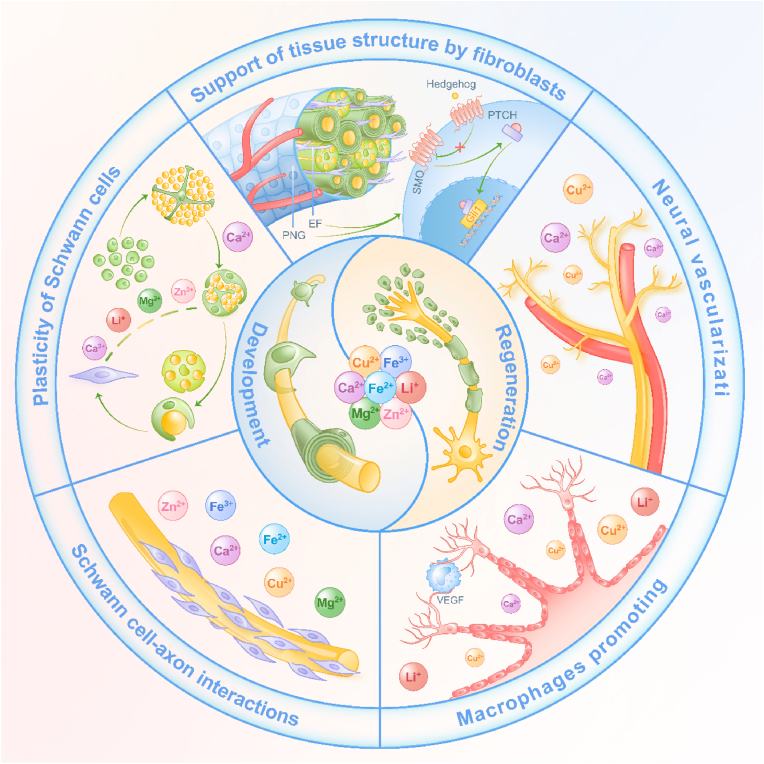
Similarities between peripheral nerve development and regeneration.

Despite significant shifts in cellular composition, metal ion regulatory profiles remain strikingly conserved between development and regeneration. Mounting evidence indicates that PNR recapitulates key developmental modules, including the reactivation of embryonic Schwann cell phenotypes, neurovascular guidance parallels, and developmental pathways like Sonic Hedgehog (Shh) (Fig. 8). A pivotal cell state, rSCs, that emerges post-injury partially reacquires embryonic progenitor traits, including stem-like properties [143]. Mirroring late developmental stages, these rSCs ensheath and guide growing axons, subsequently organizing into cellular cords that provide precise physical tracks for extension [218,219]. The Shh pathway, which governs fibroblast-mediated barrier formation in development, is reactivated to promote repair [119,220]. Similarly, the essential neurovascular coupling is reconstituted; effective axonal elongation depends on the permissive substrate of regenerating vasculature, re-establishing functional interfaces [221,222]. Moreover, macrophages consistently serve as angiogenic regulators across life stages, guiding vascular network formation in embryos and promoting neovascularization after injury to construct the requisite nutritional and guidance niche for regeneration [223,224].

Given the evolutionarily conserved regulatory roles of metal ions, we propose a “development-inspired tissue engineering” strategy: to design spatiotemporally programmed, multi-ion-loaded scaffolds that recapitulate developmental signaling networks and direct cellular behavior. Empirical support is now emerging from multiple frontiers. Self-evolving neural scaffolds that recapitulate embryonic bioelectricity achieve repair outcomes comparable to autografts, while stem cell-derived organoids engineered to emulate developmental programs enable rapid regeneration of critical bone defects [225]. These breakthroughs underscore the broad potential of simulating developmental microenvironments. As pivotal bridges linking development and regeneration, metal ions leverage their conserved properties to reconstruct missing spatiotemporal signals and re-establish critical developmental nodes. This approach functionally connects developmental biology with regenerative medicine, potentially compensating for the “spatiotemporal gap” in regeneration and offering a novel pathway for precision nerve repair.

## 6 Peripheral nerve engineering with metal ion-loaded scaffolds

**Fig. 9:**
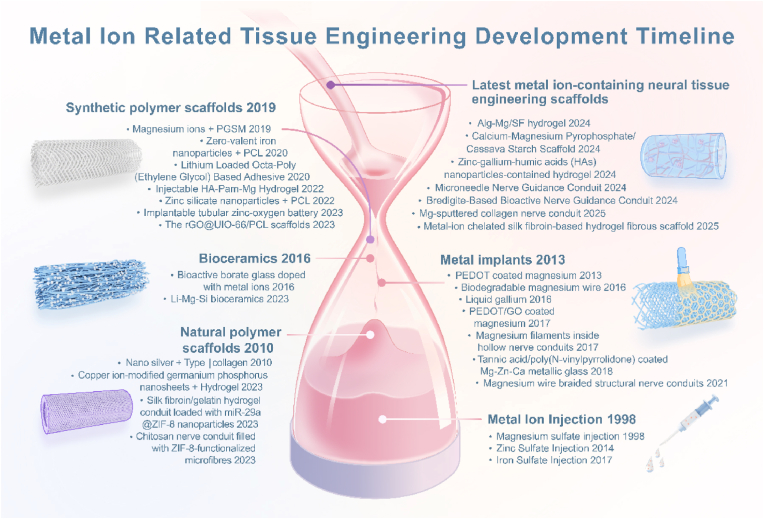
Evolution timeline of metal ion-integrated neural tissue engineering scaffolds.

The therapeutic use of metal ions in nerve regeneration, initiated in 1998 with the neuroprotective effect of localized magnesium, has undergone a critical paradigm shift [226]. This shift was necessitated by the inherent drawbacks of free ions, particularly their uncontrolled systemic diffusion causing off-target effects and toxicity [227]. Consequently, the field pivoted to incorporating metal ions into tissue engineering scaffolds, a strategy that allows for precise spatiotemporal control over ion release and reconstruction of the extracellular microenvironment [228]. This evolution is chronicled by key developments: the 2010 integration with synthetic polymers [228]; the 2013 creation of coated biodegradable implants [229]; the 2016 advent of multi-ion bio-ceramics [230]; and the 2019 design of ion-uniform synthetic polymers [231]. Current cutting-edge research has yielded intelligent, multi-ion scaffold systems that operate safely and controllably, with efficacy now rivaling that of autologous transplantation (Fig. 9) [232,233]. The following section explores these applications, focusing on the mechanism by which scaffold-based delivery enhances nerve regeneration.

### 6.1 Fabrication of metal-ion functionalized tissue engineering scaffolds

Electrospinning currently dominates the fabrication of metal ion-loaded conduits, leveraging its nanoscale resolution and microstructural design capabilities to achieve uniform ion dispersion and gradient release [234,235]. While 3D printing provides distinct advantages in macrostructural adaptability, its progress has been constrained by limited precision in microscale metal ion loading [236,237]. New pathways are emerging through high-resolution printing and hybrid methods [236], such as integrating 3D-printed scaffolds with electrospun coatings, to enable sophisticated ion integration [238]. This section will explore both the established use of electrospinning and the burgeoning potential of these advanced 3D printing techniques.

#### 6.1.1 Electrospinning for metal ion-functionalized tissue scaffolds

**Fig. 10:**
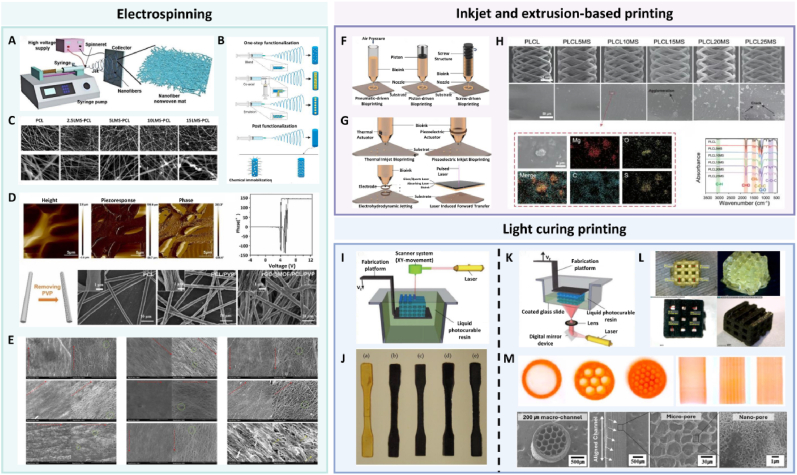
**Fabrication technologies of metal ion-integrated tissue engineering scaffolds.** (A) Schematic diagram of classical electrospinning. The image in (A) is derived from Fig. 3A of Li et al. [242] (B) The main methods for incorporating functional components into electrospun fibers. The image in (B) is derived from Fig. 2 of Liu et al. [243] (C) Electrospun fibers containing li-mg-si bioceramics. The image in (C) is derived from Fig. 2A of Sun et al. [14] (D) Piezoelectric rGO@MOF/PCL scaffolds manufactured by electrospinning. The image in (D) is derived from Fig. 2 of Yao et al. [244] (E) PHBV-magnesium oleate directional nanofibers manufactured by electrospinning. The image in (E) is derived from Fig. 2 of Ramburrun et al. [245] (F,G) Schematic diagram of inkjet and extrusion-based printing. The image in (F) and (G) are derived from Fig. 5, Fig. 12 of Gu et al. [246] (H) Extrusion-printed scaffolds with sustained mg^2+^ release. The image in (H) are derived from Fig. 2 of Zhang et al. [247] (I, K) Schematic diagram of SLA and DLP. The image in (I) and (K) are derived from Fig. 5 of Song et al. [248] (J) In situ generation of resin containing silver nanoparticles with SLA. The image in (J) is derived from Fig. 1 of Taormina et al. [249] (L) DLP-fabricated scaffolds incorporating functional nanoparticles. The images in (L) are derived from Fig. 1 of Kordi et al. [250] (M) Digital light processing printed biodegradable hydrogel-based multichannel nerve conduits. The image in (M) are derived from Fig. 4 of Maeng et al. [251].

Electrospinning is a mainstay in fabricating tissue engineering scaffolds for peripheral nerve repair (Fig. 10A–C) [239,240]. Nevertheless, electrospun NGCs, while capable of bridging peripheral nerve defects, generally yield only 50-60% of the regenerative efficacy of autologous nerve grafts. Their performance is particularly inferior in fostering a supportive microenvironment, including the recruitment of Schwann cells and control of inflammation [241]. Integrating multifunctional metal ions may address these limitations by more fully mimicking the regenerative niche provided by autologous grafts.

The fabrication of Li-Mg-Si/PCL composite NGCs was achieved through electrospinning [14,211,241,244,252]. Initially, Li-Mg-Si bioceramic powder was synthesized from precursor salts via a sol-gel process. This powder was subsequently dispersed in hexafluoroisopropanol (HFIP) by ultrasonication and mixed with a polycaprolactone (PCL) solution to yield a homogeneous electrospinning feedstock [14]. Tubular guides were then fabricated from this composite under optimized parameters. Compared to pure PCL scaffolds, the Li-Mg-Si/PCL conduits demonstrated superior regenerative efficacy, attributable to the sustained release of bioactive ions (Li^+^, Mg^2+^, Si^4+^). This ion release profile facilitated key regenerative events, including M2-like macrophage polarization and Schwann cell differentiation, thereby creating a pro-regenerative microenvironment. Consequently, while not equivalent to autografts, the composite conduits markedly improved critical regeneration metrics such as cAMP signaling, axonal growth, and myelination. This method serves as a general paradigm for preparing metal nanoparticle-incorporated electrospun scaffolds, with adaptations typically confined to solvent choice and processing details.

Metal ions can also be integrated by direct solution blending during precursor preparation, ensuring uniform dispersion in the electrospun scaffold [253]. For instance, incorporating magnesium oleate (MgOl) and N-acetyl-L-cysteine (NAC) into PHBV scaffolds promoted Schwann cell proliferation and neurite extension via Mg^2+^ release. Notably, higher additive concentrations significantly altered the electrospinning solution's properties, increasing viscosity and causing expansion. Thus, optimizing blend-based strategies must account for the significant effects of ionic additives on both biological performance and critical physical properties of the scaffold.

Moving beyond in-process blending, post-fabrication methods enable tailored metal ion integration. One sophisticated strategy constructs a dynamic matrix from methacrylated silk fibroin and acrylated bisphosphonate. This design allows for custom-shaped electrospinning and on-demand functionalization by chelating synergistic metal ions (Mg, Ag, Zn), which in implantation promoted autograft-level axonal growth and myelination [232]. Alternatively, physical methods like magnesium sputtering permit precise surface deposition, enabling controlled Mg^2+^ release without the risk of gas-induced necrosis [254]. Implantation results demonstrated morphological recovery matching autografts, with functional recovery nearing that benchmark within 8 weeks.

#### 6.1.2 Freeze/drying for metal ion-functionalized tissue scaffolds

Freeze-drying enables the fabrication of scaffolds with a porous architecture conducive to cell adhesion [254]. Utilizing this technique, we engineered bioactive NGCs by homogenously dispersing nano-silver particles within a collagen I/gelatin matrix through a controlled lyophilization process. This functionalization with silver nanoparticles markedly enhanced the scaffold's properties, endowing the NGCs with distinct regenerative advantages. Specifically, they exhibited increased laminin adsorption and supported robust nerve regeneration, characterized by enhanced myelination, improved nerve conduction velocity, and accelerated axonal regrowth. These results underscore the conduit's promise as a viable alternative to autografts [255].

#### 6.1.3 Engineering metal ion-functionalized scaffolds by 3D printing

While 3D printing excels in fabricating customized NGCs with complex, axonal-guiding architectures (Fig. 10D–I), its combination with therapeutic metal ions for peripheral nerve repair lags behind applications in other tissue engineering fields [256]. To bridge this gap, we look to advancements in bone and vascular engineering [257,258]. The latter, in particular, has achieved a breakthrough by employing metal ion doping and low-temperature coaxial extrusion to construct a scaffold with spatiotemporally defined release profiles (outer Ag^+^, inner Sr^2+^), proving highly effective in managing infection and stimulating regeneration in a bone infection model [259]. This successful paradigm, centered on achieving precise spatiotemporal control through structural design, offers a compelling and directly translatable strategy for innovating NGCs design.

#### 6.1.4 Biomimetic micro/nanostructural design: lessons from multilevel neurium and beyond

The faithful replication of native nerve topography has ascended as a preeminent paradigm for augmenting regenerative trajectories, surpassing the fabrication strategies enumerated heretofore [260,261]. Drawing inspiration from the tripartite architecture of peripheral nerves, a composite of epineurium, perineurium, and endoneurium, the “multilevel neurium-mimetic” graft (SpinMed) was architected via additive manufacturing synergistically conjoined with phase separation and gradient freeze-drying [262]. This construct effectuates efficacious neural restitution entirely bereft of exogenous growth factors, predicated solely upon a selectively protective ensheathing layer and anisotropically aligned topographical cues that choreograph oriented vascular ingression and axonal elongation.

Notwithstanding these auspicious advances, the integration of metal ions within such sophisticated multilevel biomimetic architectures remains a conspicuously underexplored frontier. Seminal investigations have substantiated the feasibility of amalgamating metal ions with elementary topographical cues, encompassing aligned Li-Mg-Si/PCL fibrous matrices [14], Zn^2+^-eluting zinc silicate nanoparticles embedded within PCL nanofibrous conduits [154], and chelated Mg^2+^/Ag^+^/Zn^2+^ reservoirs sequestered within silk fibroin scaffolds [232]. These proof-of-concept exemplars corroborate that oriented fibrous topographies co-deployed with controlled metal ion liberation are both technically attainable and therapeutically consequential. All prevailing platforms, however, remain constrained to single-lamina aligned fiber architectures that recapitulate exclusively the perineurial orientation, failing to incorporate more intricate biomimetic hallmarks such as gradient-aperture microchannels, hierarchical epineurium/perineurium/endoneurium stratification, or three-dimensionally delineated channel networks with spatial fidelity. The faithful replication of native nerve micro/nanotopography actively orchestrates cellular behavior and regenerative dynamics in a manner that conventional monolithic scaffolds cannot achieve [263].

The strategic convergence of topographical instruction and ion-mediated signaling holds profound potential to engender a synergistic “physicochemical niche” that recapitulates the developmental microenvironment with heightened verisimilitude. Forthcoming scaffold engineering endeavors should therefore aspire to the seamless amalgamation of multilevel neurium-mimetic architectures with spatiotemporally orchestrated metal ion delivery modalities, thereby bridging the critical lacuna between passive structural biomimicry and active molecular programming of the regenerative milieu.

### 6.2 Metal Ion-Loaded Scaffolds for peripheral nerve tissue engineering

The paradigm for an optimal scaffold in neural tissue engineering integrates several core characteristics: sufficient mechanical strength and structural stability, biocompatibility, excellent processability, and superior cytocompatibility [264]. Incorporating metal ions elevates these requirements, necessitating controlled, time-regulated release and, ideally, targeted and intelligently responsive delivery systems (Table 4). Consequently, biomimetic strategies that replicate the embryonic developmental microenvironment have emerged as a promising direction [264]. As metal ions are pivotal to both neural development and regeneration, they are uniquely positioned to functionalize tissue scaffolds that emulate these innate developmental processes.

**Table 4:** Summary of application strategies for tissue engineering scaffolds with metal ions.

Materials	Carriers	Metal ions	Loading methods	Releasing methods	Ionic concentration	Degradation time	Reference
**Metal implants**	PEDOT coated magnesium	Mg^2+^	/	degradation	/	/	[229]
	Biodegradable magnesium wire	Mg^2+^	/	degradation	/	/	[265]
	PEDOT/GO coated Mg	Mg^2+^	/	degradation	/	/	[266]
	Magnesium filaments placed inside poly(caprolactone) nerve conduits	Mg^2+^	/	degradation	/	Significantly degraded after 6 weeks.	[267]
	tannic acid/poly(N-vinylpyrrolidone) coated Mg-Zn-Ca metallic glass ribbon	Mg^2+^, Ca^2+^, Zn^2+^	/	degradation	/	/	[144]
	Fibroin (SF) and magnesium filament (S/Mg) composite structure(S/MG-SF/CS)	Mg^2+^	Magnesium filaments were braided into an inner layer of NGC	degradation	S/MG-SF/CS can increase Mg^2+^ concentration by about 2-3 μg/L	After 6 weeks of in vivo degradation, the SF yarn layer has almost no degradation, and the diameter of the Mg filament (0.1 mm) reduced to half	[268]
**Natural polymer scaffolds**	Tissue engineering scaffold made of nano silver and type I collagen	Ag^+^	/	degradation	During the preparation of the scaffold, 1/10 of the nano-silver solution with a volume concentration of 20,100 ppm was added	/	[255]
	Hydrogel containing germanium phosphorus nanosheets modified with copper ions	Cu^2+^	The GeP nanosheets stably trap copper ions through coordination and electrostatic attraction	degradation	The concentration of GeP@Cu was 0.5 mg mL. The hydrogel sample (0.5 mL) was soaked in 5 mL salt water and incubated at 37 °C for 21 days. At 21 d, the concentration of copper ion does not exceed 0.15 mM	The biohybrid hydrogels containing GeP@Cu retained 90% of their mass on day 1 and 38% on day 3 after enzymatic degradation	[269]
	silk fibroin/gelatin hydrogel conduit loaded with miR-29a@ZIF-8 nanoparticles	Zn^2+^	Zinc ions are included in the MOF material by means of coordination	degradation	When the Zn^2+^ concentration reached a stable level on the fifth day, hydrogel catheters containing 0.06%, 0.08%, and 0.1% miR-29a@ZIF-8 released 0.551, 0.74, and 0.84 mg/L of Zn^2+^, respectively.	/	[270]
	Chitosan nerve conduit filled with ZIF-8-functionalized microfibres	Zn^2+^	Zinc ions are included in the MOF material by means of coordination	degradation	/	/	[271]
	Alg-Mg/SF hydrogel	Mg^2+^	The electrostatic interaction and dynamic coordination between Alg-BP and cations led to the swift formation of the primary hydrogel network	degradation	About 14% of the magnesium ions were released in 14 days	/	[171]
	Amorphous Calcium-Magnesium Pyrophosphate/Cassava Starch Scaffold	Mg^2+^, Ca^2+^	Water molecules formed hydrogen bonds with pyrophosphate at varying strengths and bind to the surface of metal ions	degradation	AMCP/CS scaffolds had an explosive release of Ca and Mg ions at 1 day and tended to be a stable release after 5 d.	AMCP/CS scaffolds were degraded after 21 days in PBS.	[272]
	nanocomposite hydrogel containing pH-responsive zinc-gallium-humic acids (HAs) nanoparticles	Zn^2+^, Ga^3+^	HAs can bind cationic metals.	pH sensitivity may enable HAs to release Zn^2+^ and Ga^3+^ according to the pH value in the fracture microenvironment.	The release of Zn^2+^ from the Zn@HAs@HN hydrogel was faster than that of Ga^3+^.	The degradation rates of the Zn@HAs@HN hydrogel and Zn-Ga@HAs@HN hydrogel were similar, degrading to 30–40% on day 21.	[152]
	Mg-Sputtered collagen Nerve Conduit	Mg^2+^	Pure Mg was deposited onto the collagen scaffold by sputtering	degradation	The collagen@3 Mg sheet achieved 12.862 ± 0.250 mm within 48 h	/	[254]
**Synthetic polymer**	Zero valent zinc nanoparticles/PCL scaffold	Zn^2+^	/	degradation	/	/	[273]
	hybrid of magnesium ion (Mg2+) and poly(glycerol-sebacate-maleate) (PGSM-Mg)	Mg^2+^	Mg2+ incorporated into PGSM molecules through a complexation interaction	Ester bond hydrolysis of PGSM-Mg main chain	The cured PGSM-Mg scaffold contained 5.40 ± 0.40 mg/g of Mg^2+^, with a cumulative release of 54.8 ± 4.2% over 28 days	PGSM-Mg gradually released Mg^2+^ in PBS, and the total mass loss within 28 days was 31.0 ± 2.4%. The mass loss of cured PGSM-Mg scaffold within 28 days was 22.5 ± 3.3%.	[231]
	PHBV-magnesium oleate-N-acetyl-cysteine (PHBV-MgOl-NAC) nanofibers	Mg^2+^	MgOl was added into PHBV solution	degradation	/	/	[245]
	Zero-valent iron nanoparticles containing nanofiber scaffolds	Fe^3+^	/	degradation	Nanoparticles containing 10 wt% Fe are preferred	/	[252]
	Lithium Loaded Octa-Poly (Ethylene Glycol) Based Adhesive	Li^+^	LiCl was loaded into the adhesive system (OSSL)	degradation	The lithium chloride solution used for preparation is 0.2 gLiCl dissolved in 1 mL water. When immersed in water in vitro, lithium can be released from OSSL continuously for 14 days, with a total release of 85%. Within 14 days, only 44% of the body is released	A 5 wt% cysteamine solution can dissolve 500 μL of hydrogel in 50 min. Dissolve 20 μL hydrogel on the transected nerve in 40 min. Using a swab to accelerate dissolution can remove the hydrogel from the wound within 2 min	[274]
	Injectable HA-Pam-Mg Hydrogel	Mg^2+^	Mg2+ was encapsulated in hydrogels	degradation	The best concentration in hydrogel is 50 mM	It's related to the concentration of magnesium ions in the hydrogel. The HA-Pam-Mg-50mM hydrogel lost 22.54% and 87.5% of its original weight after 24 h and 15 days, respectively	[275]
	Zinc silicate nanoparticles-incorporated multifunctional bioactive nanofibrous scaffold	Zn^2+^	/	degradation	The PCL scaffold containing 5 wt% ZS nanoparticles has the best effect	/	[154]
	rGO@UIO-66/PCL scaffolds	Zr^4+^, Ca^2+^	Metal ions are included in the MOF material by means of coordination	degradation	/	/	[244]
	PCL scaffold containing zinc oxide	Zn2+	/	degradation	Less than 6% (i.e. 0.06 mass of Zn released normalized to initial mass of Zn in the scaffold) Zn was released from the scaffold.	/	[205]
	CuCS/Cur composite wound dressings	Cu^2+^	/	degradation	On day 5, the Cu2+ release from 1-CuCS/Cur, 5-CuCS/Cur, and 10-CuCS/Cur was measured at 21.06 ± 0.02 μg/mL, 36.08 ± 0.03 μg/mL, and 75.02 ± 0.08 μg/mL, respectively	/	[211]
	Silver Ion and Graphene Oxide-Incorporated Ethylene Vinyl Alcohol Copolymer	Ag^+^	Silver ions were doped into solution	Degradation	/	/	[253]
	PDA-Fe@PLCL Conduit	Fe^3+^	Fe3^+^ ions chelated with phenolic hydroxyl groups of dopamine derivatives on PLCL	Degradation	/	/	[276]
**Bioceramics**	Bioactive borate glass doped with metal ions	Ag^+^, Cu^2+^, Ca^2+^, Fe^3+^, Ga^3+^, Zn^2+^, Sr2+, Ce^3+^, Y^3+^	/	degradation	14 mg/mL	/	[230]
	Li-Mg-Si bioceramics	Mg^2+^, Li^+^	LMS bioceramics were prepared by electrospinning and attached to nanofiber scaffolds	degradation	/	/	[14]
	Bioactive glass doped with copper and magnesium ions	Mg^2+^, Cu^2+^	incorporating copper-doped and magnesium-doped BG into GelTA/PEG-NHS hydrogel	degradation	/	hydrogel degraded in 7 days	[277]
	Bredigite (BRT, Ca7MgSi4O16) bioceramic	Ca^2+^, Mg^2+^	BRT bioceramics were prepared by electrospinning and attached to PCL scaffolds	degradation	/	/	[182]
	Li-Ca-Si bioceramics	Li^+^, Ca^2+^	LCS bioceramic particles were incorporated into GelMA matrix	degradation	/	/	[278]

#### 6.2.1 Metal implants

**Fig. 11:**
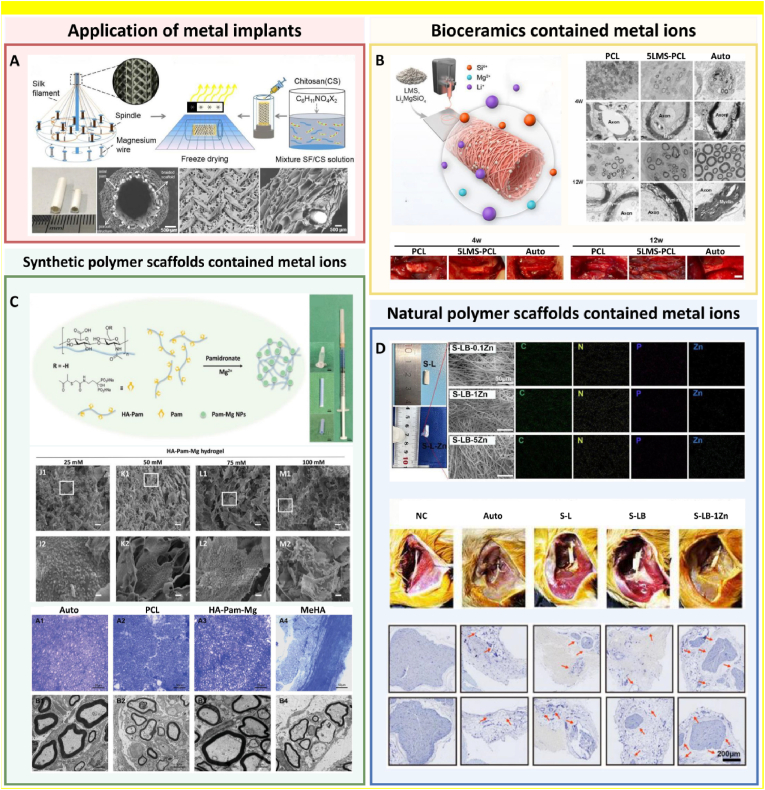
**Material taxonomies of metal ion-integrated tissue engineering scaffolds.** (A) Fibroin and magnesium filament composite structure. The images in (A) are derived from Fig. 1(B) AND (C) of Zhang et al. [268] (B) Li-Mg-Si bioceramics involving in neural engineering scaffolds. The images in (B) are derived from Fig. 7A of Sun et al. [14] (C) Injectable HA-Pam-Mg Hydrogel. The images in (C) are derived from Fig. 2 of Zhi et al. [275] (D) Silk fibroin-based hydrogel fibrous scaffold with metal-ion chelation. The images in (D) are derived from Figs. 2c and. 7g of He et al. [232].

Biodegradable metals, particularly magnesium, are promising candidates for neural tissue engineering implants due to their suitable mechanical properties and excellent biocompatibility (Fig. 11A). However, while evidence supports their role in PNR [279,280], the inherently soft nature of neural tissue poses a significant challenge for pure metal implants [267]. To circumvent this limitation, magnesium can be incorporated as internal filaments within NGCs. A more advanced strategy involves weaving these magnesium filaments with biopolymers like fibrous proteins and chitosan to form a composite conduit. Such a design not only modulates the in vivo degradation kinetics of magnesium to prolong its functional residence time but also provides enhanced spatiotemporal mechanical support. Together, these effects create a permissive physicochemical microenvironment that is crucial for effective nerve regeneration [267].

**Fig. 12:**
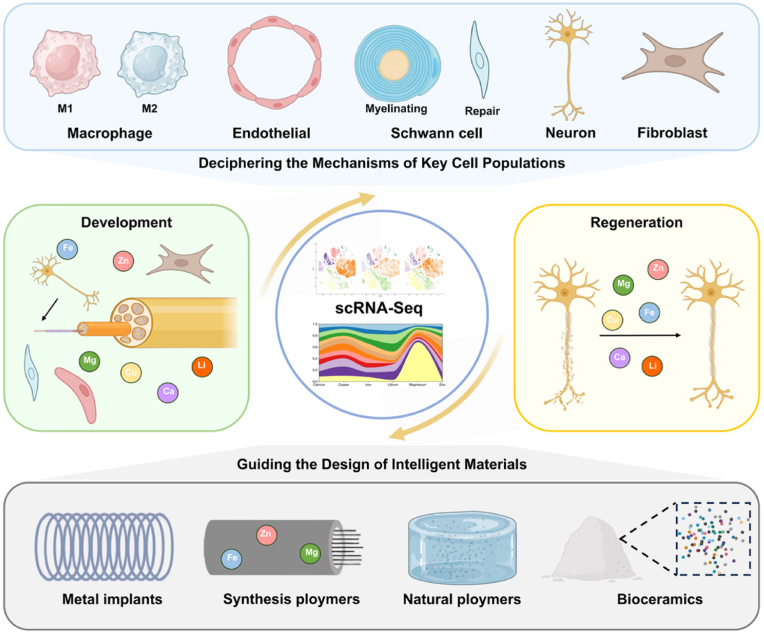
Single-cell sequencing-informed design of peripheral nerve tissue engineering scaffolds. (Created by Biorender).

The translational potential of magnesium-based implants in neural tissue engineering is significantly limited by their rapid in vivo corrosion and the associated hydrogen evolution [281,282]. A promising approach to mitigate these issues is the application of conductive polymer (CP) coatings. The first strategy involved coating magnesium with poly(3,4-ethylenedioxythiophene) (PEDOT), which was shown to control corrosion and improve cytocompatibility [229]. This approach was further optimized by doping the PEDOT matrix with graphene oxide (GO). The PEDOT/GO composite coating synergistically slowed corrosion kinetics and maintained a safe, stable local concentration of magnesium ions. Impressively, this system also demonstrates a “fail-safe” mechanism, where localized reactions at coating defects generate a durable magnesium phosphate layer that continues to impede metal ion release [266,283]. An alternative method for enhancing the performance of magnesium-based metallic glasses involves a layer-by-layer tannic acid/poly(N-vinylpyrrolidone) coating, which provides combined corrosion resistance and self-healing capability [144]. These modified surfaces significantly promoted Schwann cell viability, an effect potentially mediated by the optimized ionic microenvironment and the enhanced cell attachment facilitated by the coating's micro-roughness [284].

The future application of metal implants lies in engineered alloys that co-deliver specific metal ions and tailored mechanical properties. Nevertheless, while surface coatings can decelerate ion release, achieving fine-tuned, spatiotemporal control over this process remains elusive. This critical gap currently hinders the translation of metal implants into precision therapeutics for nerve regeneration.

#### 6.2.2 Natural polymer scaffolds

Polymer scaffolds represent a predominant strategy in neural tissue engineering, prized for their customizable morphology and properties [21]. Their porous architecture serves to host metal ions, a loading process that simultaneously tunes the scaffold's mechanical properties (Fig. 11B). Upon implantation at a nerve injury, these constructs provide critical structural support and topographical guidance, while their degradation enables the sustained release of incorporated metal ions.

Owing to their biocompatibility and structural resemblance to the native extracellular matrix, natural polymers are a mainstay in neural tissue engineering [285]. A common method to functionalize these polymers with metal ions is through the incorporation of metal nanoparticles. This is exemplified by a collagen I/nanosilver composite scaffold, where synergistic interactions improved the adsorption of adhesion proteins, leading to significantly enhanced axon regeneration in vivo [180]. Beyond their standalone use, natural polymers also serve as components in composite systems with ion-releasing rigid scaffolds. One such composite, an amorphous magnesium-calcium pyrophosphate (AMCP)/cassava starch (CS) scaffold, demonstrates the ability to release magnesium ions over an extended period, effectively fostering Schwann cell proliferation [272].

Hydrogels derived from natural polymers leverage their porous, hydrophilic, and biodegradable nature to create exemplary scaffolds for PNR. Metal ions like Cu^2+^ and Mg^2+^ can be directly loaded to exert bioeffects, and they often serve a dual role: integrating into the porous network and participating in cross-linking to enhance mechanical strength [286,287]. Capitalizing on the fact that compliant substrates better promote regeneration [288], a biodegradable GelMA hydrogel incorporated germanium phosphide (GeP) nanoplates and Cu^2+^. The Cu^2+^ modification boosted the hydrogel's conductivity, antimicrobial activity, and biocompatibility, enabling excellent antibacterial performance and significantly enhanced nerve regeneration in vivo [269].

Metal-organic frameworks (MOFs) are emerging as key additives to enhance natural polymer scaffolds for nerve repair. Incorporating ZIF-8 into silk fibroin/gelatin-tyramine hydrogels simultaneously promotes axon extension and increases hydrogel toughness [270,289]. Advancing this concept, a chitosan NGC with ZIF-8-modified fibers (CS@ZIF-8) surpassed controls in rat models, leveraging zinc ion release and structural guidance [271]. To precisely control delivery, pH-responsive zinc-gallium-humic acid nanoparticles were designed to release ions in the regenerative microenvironment's alkaline milieu, significantly accelerating Schwann cell migration [290]. Further personalization is now possible with electrospun, photocurable methacrylated silk fibroin (SFMA) fibers functionalized with acrylated bisphosphonate (AcBP), which chelates therapeutic ions (Mg^2+^, Ag^+^, Zn^2+^) for tailored nerve regeneration applications [232].

#### 6.2.3 Synthetic polymer scaffolds

Synthetic polymer scaffolds are highly advantageous for nerve regeneration due to their precisely tunable properties, including biodegradability and mechanical strength (Fig. 10M) [291]. Frequently used polymers encompass poly(ε-caprolactone) (PCL), poly(lactic-co-glycolic acid) (PLGA), polylactic acid (PLA), polyethylene glycol (PEG), polyvinyl alcohol (PVA), and poly(propylene fumarate) (PPF). A novel approach directly integrates metal ions into the polymer matrix, as seen in a poly(glycerol-stearate-maleate)-Mg (PGSM-Mg) composite. This material biodegrades appropriately while sustaining Mg^2+^ release, which significantly upregulates nerve growth factor genes in Schwann cells, enhancing their adhesion and proliferation. For polymers lacking intrinsic metal-binding sites, surface functionalization offers an alternative. A polydopamine (PDA) coating applied to PLCL scaffolds enables efficient Fe^3+^ chelation, creating a bioactive interface that directs Schwann cell behavior and facilitates nerve regeneration [276].

Synthetic hydrogels provide a versatile platform for nerve repair, largely due to their enhanced capacity for metal ion coordination, which can be strategically leveraged across application formats. As crosslinked scaffolds, systems like a bisphosphonate-Mg^2+^ hydrogel utilize ion coordination to form stable nanostructures, underpinning a controlled release profile that enhances neurite outgrowth, myelination, and functional recovery [275,292]. Translated into bioadhesives, this principle is exemplified by a lithium-ion-loaded PEG-based polymer. Its design incorporates succinyl units for safe dissolution and releases Li^+^ to significantly boost axonal regeneration and functional outcomes [274]. Beyond biochemical signaling, metal ions can modulate scaffold microarchitecture, enabling a synergistic dual-cue strategy for nerve repair. Electrospinning produces aligned nanofibers that replicate the anisotropic physical cues of the extracellular matrix to support axonal extension [138,293]. Integrating metal ions can further enhance this structural guidance.For instance, adding Ag + to an EVAL polymer precursor directly governs the microarchitecture of electrospun fibers, leading to anisotropic scaffolds that effectively direct neurite growth [253]. Future electrospinning strategies should exploit this structure-activity synergy, optimizing ion-induced fiber alignment to bridge longer nerve gaps.

Recent progress in neural tissue engineering highlights the superiority of scaffold-mediated ion delivery over free ions, achieved through tunable release kinetics and multifunctional design. This is exemplified by nanoparticles embedded in PCL nanofibers: iron nanoparticles confer sustained ion release and enhanced conductivity within a fully biodegradable system [252], while zinc silicate nanoparticles serve as a stable source of zinc and silicate ions to critically enhance Schwann cell differentiation and myelination [154]. Chiral Fe_3_O_4_ nanoparticle enantiomers, particularly the dextrorotatory form (D-Fe_3_O_4_), demonstrate chirality-selective modulation of Schwann cells through autophagy-driven p-JNK/EPHA5 pathways, promoting cellular proliferation, migration, and differentiation into remyelinating phenotypes [294]. Concurrently, MOFs are exploited for their high surface area and structural diversity. A notable example is a self-powered neural scaffold combining MOFs and rGO in a PCL matrix, which modulates the immune microenvironment via calcium/zirconium ion release to promote M2 macrophage polarization [244,295]. Further innovations include a zinc-based MOF/hydrogel composite for pro-proliferative zinc ion release [233], and a piezoelectric PCL/ZnO/rGO microneedle conduit that harnesses electrical stimulation to accelerate Schwann cell migration and axonal growth [296]. These approaches collectively underscore a shift towards sophisticated, multi-modal strategies for nerve repair.

#### 6.2.4 Bioceramics

Bioceramics are instrumental in tissue engineering for the establishment of a reparative microenvironment (Fig. 11C) [297]. Translating this potential to neural repair requires the development of innovative formulations, including composite conduits comprising ceramic powders within a polymer matrix, to achieve mechanical compliance with soft nervous tissue. Among available materials, bioactive borate glass (BBG) is a preeminent candidate due to its accelerated degradation profile relative to silicate glasses and the concomitant release of boron, which exhibits pro-angiogenic properties [298,299]. Composite scaffolds fabricated through the incorporation of metal-ion-doped BBG particles into PCL have shown considerable promise in promoting neurite outgrowth, thereby positioning them as a next-generation strategy for peripheral nerve repair [128]. The therapeutic efficacy of these constructs is constrained by a defined window, reflecting the dual functionality of boron: it potentiates cellular proliferation and may engage in synergistic interactions with co-released metal ions at low concentrations, yet it demands stringent dosage control owing to its cytotoxic effects at higher levels [300].

Accumulating evidence highlights the role of silicate-based bioceramics in fostering regeneration across various tissues [301]. A prominent example is the work of Sun et al., who fabricated a PCL NGC incorporating Li–Mg–Si (LMS) bioceramic particles. The released lithium, magnesium, and silicon ions act synergistically to promote SC proliferation, migration, and myelination, while also recruiting macrophages and inducing their polarization to the pro-regenerative M2 phenotype [14]. In a complementary approach, Xuan et al. explored a NGC derived from bredigite (BRT, Ca_7_MgSi_4_O_16_) bioceramic, which demonstrates excellent biocompatibility and sustained release of Ca^2+^, Mg^2+^, and Si^4+^ ions. These ions were found to enhance SC proliferation and myelination, as well as facilitate M2 macrophage polarization, confirming BRT's promise for peripheral nerve repair [182]. The synergistic potential of bioceramic-derived ions extends to interactions with other bioactive factors. Zhang et al. designed an electrospun wound dressing that co-delivers copper-doped calcium silicate (CuCS) and curcumin (Cur). This system releases a bioactive Cu^2+^-Cur chelate that potently upregulates neural-related factors and angiogenesis in hair follicle cells, thereby supporting the development of a mature neuronal network [211].

### 6.3 Single-cell transcriptomics-driven biomaterial manufacturing: from atlas to scaffold

The discovery of evolutionarily conserved, metal ion-mediated regulatory mechanisms via single-cell transcriptomics has established a new paradigm for neural scaffold design. This insight moves the field beyond traditional empirical approaches and toward a “cell atlas-informed manufacturing” framework. Guided by this framework, we can now engineer scaffolds that precisely mimic the spatiotemporal dynamics of ion signaling found in the native nerve regenerative microenvironment (Fig. 12).

Single-cell transcriptomic profiling has uncovered highly specialized response patterns among distinct cellular subpopulations in the peripheral nerve injury microenvironment. Recent studies demonstrate that dedifferentiated Schwann cells secrete high levels of sFRP1, which acts as a critical “inflammatory-regenerative switch”; targeted inhibition of sFRP1 significantly diminishes macrophage infiltration and promotes axonal regeneration [302]. Furthermore, in age-impaired nerve repair models, a specific Runx2^+^ Schwann cell subpopulation pathologically persists owing to disrupted stress granule homeostasis [302]. Intervention through partial reprogramming effectively restores the cellular dynamics of these cells, reinstating their capacity for myelination. These findings collectively validate a systematic regenerative medicine framework: “single-cell atlas - key target - functional intervention."

Beyond genetic or pharmacological approaches, which lack the spatiotemporal precision required for clinical nerve repair, the integration of single-cell data offers a transformative path. This integration directly translates “cellular response profiles” into “material design instructions.” Single-cell RNA sequencing provides the initial blueprint by defining cellular subsets and their molecular signatures, thereby informing the creation of targeted delivery systems [303,304]. Moreover, incorporating cell-cell communication and trajectory inference analyses allows for the quantification of cellular dynamics, which facilitates the engineering of biomaterials with biomimetic topographies and gradients. The temporal dimension of repair is addressed through pseudotemporal ordering, identifying critical phases for intervention and guiding the development of adaptive, “smart” materials [305,306]. This foundational understanding is further strengthened by cross-species comparative analysis [307,308], which confirms the evolutionary conservation of key pathways and significantly enhances the predictive accuracy for clinical translation and personalized medicine.

The defined pathway from single-cell atlases to functional manufacturing represents a precision therapeutic strategy rooted in biological principles. This pipeline begins by constructing multi-temporal regeneration atlases using single-cell technologies, which identify key cellular subpopulations, molecular targets, and spatiotemporal dynamics that drive optimal repair. These insights are then translated into concrete design parameters: bioactive factor release kinetics guided by pseudotemporal trajectories, functional agents selected based on critical ligand-receptor pairs, and material microarchitecture informed by spatial co-localization patterns. Guided by these parameters, intelligent biomaterials are rationally manufactured to precisely mimic and steer endogenous repair. Finally, the mechanisms and efficacy of these constructs are re-evaluated with single-cell technologies, closing the iterative loop from biological discovery to engineered fabrication. This self-refining cycle accelerates regenerative medicine toward unprecedented precision and personalization.

## 7 Metal ion-based nerve scaffolds: clinical translation bottlenecks and cross-field paradigms

Notwithstanding significant progress, the clinical translation of metal ion-based tissue engineering scaffolds faces persistent challenges. These include achieving precise control over ion release kinetics, balancing bioactivity with cytotoxicity, and developing delivery strategies suited to complex clinical environments.

### 7.1 Cross-field paradigms: clinical translation of metallic biomaterials

Metallic biomaterials have achieved notable clinical translation in orthopedics, cardiovascular medicine, and wound healing. In orthopedics, magnesium-based alloys (e.g., MgYREZr, Mg-Ca-Zn) have demonstrated safety, efficacy, and bone-mimetic mechanical properties in clinical trials [309], securing market approval from China National Medical Products Administration markets (NMPA), European Conformité Européenne (CE), and Korea Ministry of Food and Drug Safety (KFDA). In cardiovascular applications, magnesium alloy stents like Magmaris hold CE marking; they degrade completely within 12 months, avoiding long-term complications, with 36-month follow-up data showing no restenosis [310]. For wound healing, silver-based dressings achieve dual antibacterial and healing-promoting functions through controlled silver ion release, with 123 products currently approved by the United States Food and Drug Administration (FDA) and a market size reaching $570 million [311].

### 7.2 Peripheral nerve microenvironment specificity: translational challenges of metallic ion scaffolds

The application of metallic ion scaffolds, though clinically established in orthopedic and cardiovascular fields, faces significant translational challenges in peripheral nerve repair, rooted in three interconnected aspects. Firstly, the mechanical compliance required by the flexible and dynamically stressed nerve environment is not met by conventional stiff metal scaffolds [312]. This mismatch risks interface destabilization and disrupts neurite outgrowth. Secondly, the neurotoxic susceptibility of neural cells is heightened, as they tolerate only a very narrow concentration range of metal ions [313]. Thirdly, the unique physiology of the blood-nerve barrier poses a distinct challenge [314]; it tightly regulates molecular transit, which adversely affects ion distribution and clearance, making local concentration control precarious and potentiating long-term toxicity.

These challenges are compounded by foundational differences in system biology between neural repair and the more mature applications of metal ions in orthopedics and cardiovascular contexts. While bone and vascular healing occur in relatively uniform cellular settings, peripheral nerve regeneration depends on the exquisitely coordinated interaction of a diverse cellular cast: dedifferentiated and myelinating Schwann cells, polarized macrophages, specialized fibroblast subpopulations, and regenerating axons. Each cell type responds to ionic signals in a temporally distinct manner. Additionally, the signaling landscape governing neural repair is markedly more intricate, necessitating the stage-specific and spatially localized activation of pathways such as Wnt/β-catenin, PI3K/Akt, and MAPK by particular metal ions. The requirement for such fine-tuned four-dimensional (temporal + spatial) control to direct axonal growth via chemical gradients constitutes a far greater regulatory hurdle than the broader osteogenic or angiogenic cues sufficient elsewhere. Consequently, it is this extreme complexity of the microenvironment and the demand for precise spatiotemporal signaling that limit the direct transfer of existing ion-delivery approaches and underline the rationale for a development-inspired framework that mimics embryonic ionic regulation.

### 7.3 Synergistic strategies: clinical translation of metal ion-integrated nerve guidance conduits

Peripheral nerve repair has seen the clinical implementation of regulatory-approved NGCs composed of collagen, PLCL, PGA, and chitosan across multiple jurisdictions including the United States (FDA), Europe (CE), and China (NMPA) (Table 5). Nonetheless, these conduits primarily function as passive guides, and the absence of bioactive signaling has resulted in limited therapeutic success, restricting their broad application [315]. The incorporation of bioactive metal ions addresses this void by actively engaging with the regeneration process, transforming a structural scaffold into a therapeutic device. Evidence from animal studies supports the promise of this strategy for improving regenerative outcomes. The design principles, fabrication techniques, and experimental validation supporting these findings are thoroughly detailed in Chapter 6 of this review, titled “Peripheral Nerve Engineering with Metal Ion-Loaded Scaffolds."

**Table 5:** Currently government-approved, commercially-available neural scaffold devices.

Product name	Material	Gap	Degradation
**Neurotube®**	Polyglycolic acid (PGA)	2.0–4.0 cm	3 months
**NeuroGen®**	Collagen type I	0.5–1.7 cm	36–48 months
**NeuroFlex™**	Collagen type I	2.5 cm	4–8 months
**NeuroMatrix™**	Collagen type I	2.5–3.0 cm	4–8 months
**AxoGuard Nerve Connector®**	Porcine small intestinal submucosa (SIS)	0.5–1.0 cm	3 months
**Neurolac®**	Poly (DL-lactide-ε-caprolactone) (PLCL)	2.0 cm	16 months
**SaluTunnel™ Nerve Protector**	Polyvinyl alcohol (PVA)	4.0–6.35 cm	Non-degradable
**Salubridge™ Nerve Cuff**	Polyvinyl alcohol (PVA)	4.0–6.35 cm	Non-degradable
**Avance® Nerve Graft**	Decellularized ECM derived from donated cadaveric nerve	2.5–3.0 cm	N/A
**NeuroWrap™**	Collagen type I	2–4 cm	36–48 months
**NeuroMend™**	Collagen type I	0.9–2.5 cm	4–8 months
**NeuraGen® 3D**	Collagen type I, glycosaminoglycan	2.5 cm	4-8 months
**Nerbridge®**	Polyglycolic acid (PGA), collagen	2.5 cm	3 months
**Reaxon®**	Chitosan	1.0 cm	74-77 weeks
**Jiangsu Yitong® “peripheral-nerve repair graft”**	Chitosan, chitin, gelatin and poly(glycolide-co-lactide) (PGLA)	≤3 cm	3 months
**Shenqiao®**	Decellularized human nerve	1–5 cm	N/A
**Beijing Tianxinfu® “artificial nerve sheath tube”**	Collagen type I	≤2 cm	3–6 months

Metal ion-based scaffolds have demonstrated significant potential for modulating the nerve regeneration microenvironment. Nevertheless, in the specific context of peripheral nerve repair, they exhibit a set of critical limitations. These constraints not only curtail the effectiveness of current strategies but also hinder the clinical translation of this promising technology.

The incorporation of bioactive metal ions into clinically approved NGCs presents a pragmatic translational strategy. This approach synergizes the validated safety profile of existing devices with the therapeutic potential of ionic cues, thereby generating a superior class of nerve repair technology. It is designed to overcome prevailing limitations while simultaneously ensuring a streamlined pathway toward regulatory approval and clinical deployment.

## 8 Breaking through clinical translation bottlenecks: existing challenges and future advanced pathways

### 8.1 Toxicity and metabolic challenges of metal ions

While metal-based implants have achieved significant clinical success in orthopedics and cardiovascular medicine, concerns are growing about their potential neurotoxicity with prolonged use. These concerns are especially acute in peripheral nerve repair, where the unique blood-nerve barrier, despite its protective function, may also sequester and accumulate metal ions, thereby exacerbating their toxic effects. Consequently, applying metal ion-based therapies for peripheral nerve regeneration necessitates a more cautious strategy, mandating a thorough evaluation of their local and systemic toxicity, metabolic kinetics, and tissue distribution.

Under physiological conditions, most metal ions in serum are present at trace baseline concentrations and are maintained within a relatively stable homeostatic equilibrium [316]. However, metallic devices implanted in the body may continuously release ions into the systemic circulation due to factors such as the material's inherent corrosion characteristics, patient-specific variables, and surgical procedural considerations. Notably, peripheral nerve injuries frequently occur in mechanically demanding regions such as the craniomaxillofacial area and extremities. The biomechanical milieu during daily activities (tensile, frictional, and compressive stresses) can exacerbate micromotion and interfacial instability at the implant–tissue interface, thereby potentiating abnormal metal ion release [317].

To address these challenges, peripheral nerve tissue engineering is pivoting toward more controllable strategies: integrating metal ions or their nanoparticles into biodegradable polymer matrices. While directly loading free metal ions is simple, it often results in poorly controlled release kinetics. This can cause an initial burst that transiently exceeds local safety thresholds and promotes systemic dissemination. In contrast, metal nanoparticles act as sustained ion reservoirs, enhancing bioavailability, prolonging therapeutic effect, and allowing precise release modulation [318]. However, nanoparticles introduce new complexities: parameters such as size, morphology, chemical composition, surface properties, dissolution rate, and aggregation propensity critically influence their biodistribution, clearance pathways, and long-term retention risks, all of which must be comprehensively considered during design [319,320].

The adverse effects of excessive metal ions on cellular and systemic homeostasis are a critical concern. While appropriate concentrations can positively regulate cells within the neural microenvironment and support regeneration, supra-physiological levels reveal a dose-dependent duality. Excess ions directly induce cytotoxicity, characterized by inflammatory cascade activation, suppressed cell proliferation and migration, and oxidative stress-driven reactive oxygen species generation, ultimately leading to cell damage or death [321]. Furthermore, free ions can disseminate via circulation or interstitial fluid, accumulating in distal organs such as the kidneys, liver, and brain and provoking systemic toxic responses [322].

### 8.2 Limitations in current metal ion-incorporated neural tissue engineering

#### 8.2.1 Knowledge gaps in metal ion-mediated peripheral nerve development and regeneration

Recent advances have highlighted the developmental heterogeneity of key cellular populations in peripheral nerves, demonstrating that phenotypically similar cells, such as epineurial versus endoneurial macrophages and lineage-distinct fibroblasts, possess unique functional attributes [91,132]. In contrast, the study of metal ions in development remains largely focused on axonal growth and Schwann cell transitions. Consequently, the roles of metal ions across these highly heterogeneous non-neuronal subpopulations constitute a significant knowledge gap.

A parallel knowledge gap exists in regeneration studies. Although omics technologies have identified refined cellular subpopulations (e.g., distinct Schwann cell states and M2 macrophage subtypes), the regulatory functions of metal ions within these populations await systematic investigation [136,233]. Moreover, in pathological contexts like chronic injury, excessive fibroblast activation and subsequent fibrosis demand particular attention [136]. Compounding this issue, the concentrations of metal ions used in experiments are largely based on in vitro data, creating a disconnect from clinical reality because direct measurement of in vivo concentrations remains technically difficult. Our integration of single-cell omics offers a predictive framework for in vivo effects, but conclusive validation through comprehensive in vivo studies is essential.

A formidable theoretical and methodological schism persists in our fundamental grasp of metal ion dynamics choreographing peripheral nerve regeneration. From a theoretical purview, notwithstanding the progressive elucidation of monovalent ionic contributions, the labyrinthine multi-ion competitive and synergistic interplay transpiring within the in vivo regenerative milieu remains conspicuously underexplored. Moreover, integrative theoretical paradigms that mechanistically conjoin dynamic ionic fluxes with mechanotransductive and bioelectrical signaling cascades across the discrete spatiotemporal phases of neural repair have yet to crystallize into coherent models.

Ameliorating these conceptual deficits mandates parallel strides in technical sophistication. Contemporary investigative methodologies remain overwhelmingly anchored to static endpoint analyses or reductionist in vitro approximations. An exigent unmet need exists for noninvasive, spatially resolved, real-time trace-metal-tracking platforms capable of resolving discrete ionic species within the living neural interstitium with subcellular precision. The deployment of such transformative instrumentation will prove indispensable for constructing high-resolution cartographies of the dynamic ionic microenvironment, furnishing empirical corroboration for nascent theoretical frameworks, and catalytically propelling metal ion-orchestrated precision neurotherapeutics from conceptual infancy toward translational maturity.

#### 8.2.2 The limitations of scaffold design approaches

The clinical translation of metal ions for nerve repair is significantly challenged by their remarkably narrow therapeutic windows [323]. Magnesium ions illustrate this critical balance: they support axonal regeneration at appropriate concentrations but provoke neuronal apoptosis via calcium dysregulation and oxidative stress at elevated levels [324]. Consequently, conventional uniform-release scaffolds are fundamentally inadequate, as they fail to adapt to the dynamic biological needs of regeneration, such as varying concentration requirements across different healing phases and cell types. Therefore, next-generation smart biomaterials are being developed with phase-responsive delivery systems that dynamically adjust ion release. This new paradigm, which entails a shift from static diffusion to spatiotemporally controlled release, is designed to enhance therapeutic efficacy and reduce potential neurotoxicity.

#### 8.2.3 The limitations of scaffold fabrication approaches

Current fabrication techniques achieve uniform ion distribution for near-linear release and surface modifications that merely shift release kinetics [229,254]. Current passive release systems rely on Fickian diffusion or bulk material degradation. This mechanism typically results in an initial burst release followed by a gradual decline in release rate. Moreover, conventional fabrication methods such as blending or dip-coating lack the spatial precision needed to establish defined ion gradients, thereby preventing site-specific dosing patterns such as a proximo-distal concentration decrement. Once implanted, the release profile becomes fixed and cannot be dynamically adjusted in response to changing biochemical cues during repair. Consequently, the regenerative microenvironment receives a static signal rather than a temporally adaptive one.

To overcome the limitations of passive diffusion in conventional scaffolds, next-generation designs are shifting toward systems with spatiotemporal controllability governed by precise physicochemical mechanisms. A key strategy involves harnessing the fluctuating pathophysiological signals of the nerve injury microenvironment as endogenous molecular switches. For instance, localized acidosis resulting from ischemia can be targeted using pH-sensitive metal-ligand coordination bonds, where acidic conditions trigger ligand protonation and bond dissociation to enable on-demand ion release [290,325]. Similarly, to address oxidative stress during the acute injury phase, ROS-responsive linkers can be incorporated into the polymer backbone; high ROS levels induce cleavage or degradation, releasing metal ions precisely when antioxidant and immunomodulatory effects are most needed [326]. Beyond endogenous cues, exogenous physical fields offer non-invasive remote control by transducing external energy into localized chemical or mechanical signals. Examples include using ultrasound-induced cavitation or piezoelectric charge accumulation to disrupt carriers or generate acoustic-thermal effects for ion release, while concurrently providing electrical stimulation [327]. Integrating such physicochemical mechanisms with structurally dynamic technologies like 4D printing transforms the nerve conduit from a static scaffold into an intelligent device capable of sensing and synchronizing with the regenerative cascade. 4D printing technology transcends this limitation by facilitating the creation of scaffolds capable of on-demand reconfiguration in response to specific physiological stimuli, thereby allowing for sophisticated, multi-stage concentration control [328]. Our prior work exemplifies this, having developed a 4D-printed shape-memory conduit that directs neural regeneration by undergoing programmed morphological changes to modify its surface microtopography in the in vivo environment [9].

Furthermore, the integration of metal ions into biomaterials often leads to unpredictable changes in their physical properties. A representative case is electrospun materials, in which the incorporation of metal nanoparticles directly alters fiber diameter [273]. Specifically, increasing zinc nanoparticle concentration reduces PCL fiber diameter, while higher iron nanoparticle concentrations enlarge it [252]. Notably, zinc silicate concentrations produce no diameter change under identical conditions [154]. We posit that differences in nanoparticle electrical conductivity, which alter the solution's electrospinning properties, underpin these morphological outcomes. This variability poses a significant manufacturing challenge for multi-ion strategies aimed at synergistic nerve regeneration, where coordinating ideal ion concentrations with optimal scaffold architecture becomes complex.

### 8.3 Future advanced pathways: multidisciplinary collaborative strategies for clinical translation breakthroughs

To overcome these limitations, a trans-disciplinary “mechanism-driven discovery - dynamic scaffold design - precision manufacturing” pipeline must be established, creating an unbroken translational chain that links molecular insights to clinically proven nerve function and delivers a true bench-to-bedside leap.

**Fig. 13:**
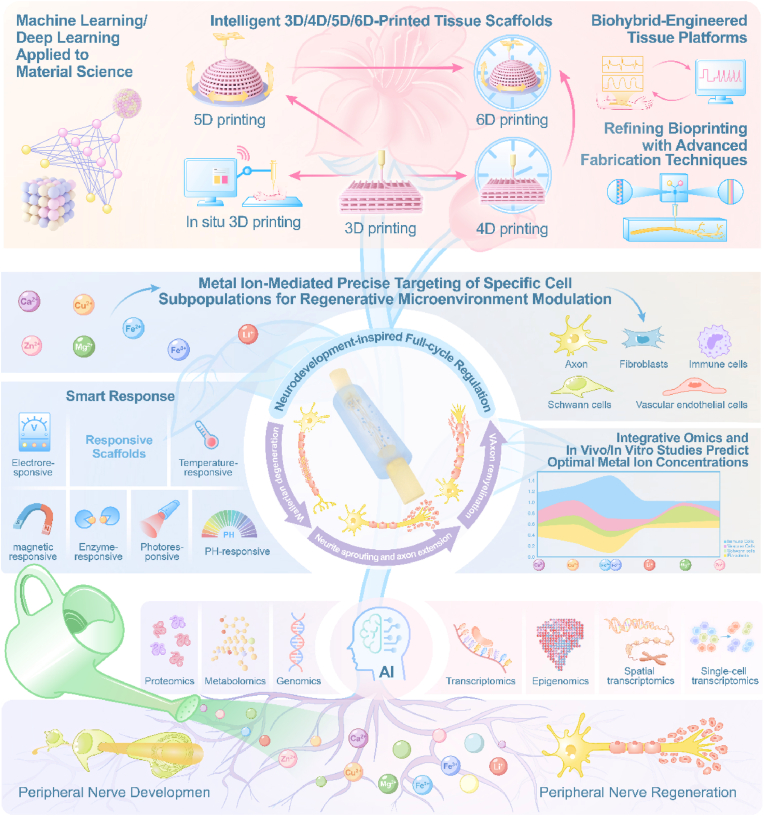
Forward-looking strategies for designing and manufacturing metal ion-loaded neural tissue engineering scaffolds.

As summarized in Fig. 13, the development of metal ion-incorporated peripheral nerve scaffolds follows a structured progression, metaphorically akin to a flowering plant. Here, fundamental biological mechanisms serve as the root, precision ion delivery systems as the vital sap, smart scaffold technologies as the supporting foliage, and the integration of advanced manufacturing with machine learning as the culminating flower. This schema demonstrates that cultivating the next generation of neural regenerative therapies is crucially dependent on the symbiotic interaction of biological knowledge, material innovation, and technological advancement.

#### 8.3.1 AI-driven deep omics analysis: elucidating the mechanisms of peripheral nerve development and regeneration

The intricate biology of the PNS continues to present persistent knowledge gaps, despite technological advances. Our prior validation of the concordance between single-cell computational predictions and empirical data underscores the potential of a unified omics approach. Integrating bulk and single-cell transcriptomics, spatial mapping, proteomics, and metabolomics across species and temporal contexts will empower the systematic construction of a high-dimensional omics atlas. This resource will elucidate the precise regulatory mechanisms by which metal ions engage specific cellular subtypes and their associated genetic networks [329]. Sophisticated machine learning algorithms will then distill this atlas into species-specific, temporally calibrated dose-response models [330], ultimately converting mechanistic understanding into quantitative scaffold design specifications for ion release and distribution.

#### 8.3.2 Biomimetic intelligent design: “developmentally-inspired” whole-cycle dynamic regulation strategy

To build upon deepening mechanistic insights, next-generation scaffolds must supersede static delivery paradigms in favor of autonomous feedback-loop operation. A transformative goal is the engineering of integrated systems capable of ‘sensing-response-adaptation’. Such systems would utilize dynamic materials to detect microenvironmental signatures (e.g., pH, enzymatic activity) and subsequently self-modulate metal ion release profiles to match evolving regenerative phases [[331], [332], [333], [334]]. Coupled with molecularly targeted modifications that precisely engage discrete cell subtypes and core signaling pathways (MAPK, PI3K/Akt), these scaffolds would constitute a dynamically tunable regulatory network, effectively reconstituting the resilient developmental program that drives robust nerve growth.

#### 8.3.3 Advancements in precision manufacturing: synergistically driving neural niche remodeling with metal ions

Leveraging breakthroughs in biomedical engineering, neural repair is progressing towards unprecedented precision. 3D printing enables the layer-by-layer fabrication of nerve scaffolds with biomimetic microstructures, whose topological design can direct the spatial pattern of ion release [335]. Building upon this, 4D printing introduces a temporal dimension by incorporating stimuli-responsive materials, allowing scaffolds to autonomously change shape and modulate metal ion release according to the stage of repair [9]. Looking forward, 5D/6D printing further augments precision manufacturing by incorporating additional parameters, such as chemical and biological signals, potentially enabling the integration of complex metal ion metabolic networks for achieving superior, functionally adaptive regeneration [336].

Emerging bioprinting strategies offer unprecedented control over neural regeneration. In situ bioprinting allows the topographical deposition of metal ion-functionalized bioinks directly at the lesion site, enabling immediate modulation of the injury niche [337]. Convergence with Biohybrid Engineered Tissue (BHET) systems, which fuse living tissues with biomedical electronics, facilitates continuous multi-parametric biofeedback, permitting real-time ionic adjustment and dynamic species substitution to fulfill precision therapeutic regimens [338]. On the materials front, dynamic bioinks exploit reversible covalent and supramolecular chemistries to create networks for controlled ion elution, while microfluidic bioprinting enables the fabrication of compartments with distinct ion-release profiles for sophisticated spatiotemporal programming [339]. A prevailing challenge of low post-printing cell viability may be mitigated by incorporating pro-survival metal ions such as lithium, as evidenced by recent studies [340].

AI-driven material design can analyze vast amounts of biomaterial data to establish predictive models of “ion concentration-cell response-functional output,” optimizing the combination and release kinetics of metal ions in scaffolds [341]. Reinforcement learning algorithms empower the adaptive adjustment of printing process parameters, ensuring the stable formation of complex ion gradients.

The growing convergence of advanced assessment methods delineates a cautious yet promising translational pathway. Innovations in multi-omics technologies provide powerful tools for systematically elucidating toxicity with molecular precision. Furthermore, interdisciplinary approaches, such as mathematical modeling for toxicity prediction, virtual cell systems for simulating ion effects, and machine learning for biomarker discovery from high-throughput data, are substantially accelerating the field [308,342]. Together, these methodologies support a multidisciplinary clinical translation roadmap for neural regeneration, built upon an integrated triad of AI-driven omics decoding, biomimetic intelligent design, and precision manufacturing. This combination creates a self-reinforcing cycle: deeper biological insights inform smarter material designs, which are realized through advanced fabrication, in turn generating new data that refine biological understanding. Such an integrative framework promises to shift the paradigm from static implants toward dynamic regenerative ecologies, in which scaffolds act as active, communicative partners in healing. Achieving this vision will require fostering unprecedented collaboration across computational biology, materials science, and clinical neurology to translate technological advances into safe, effective, and accessible therapies for peripheral nerve repair.

## 9 Conclusions

Despite advances in therapeutic strategies, peripheral nerve regeneration continues to face fundamental challenges, including slow regeneration rates, imprecise axonal guidance, and a dysfunctional microenvironment. Metal ions, as endogenous signaling agents, can be harnessed to address these issues through multi-targeted and phase-specific regulation of key cellular processes. Their roles encompass orchestrating Schwann cell dynamics, modulating immune responses, guiding stromal remodeling, and promoting axonal growth throughout development and repair. This review systematically synthesizes the spatiotemporal regulatory mechanisms of metal ions and proposes a novel “development-inspired” framework for neural tissue engineering. By recapitulating embryonic signaling cues through controlled ion delivery and leveraging advanced fabrication technologies, this paradigm enables the design of intelligent scaffolds that can dynamically regulate the regenerative niche. Moving forward, research should focus on three key directions: artificial intelligence-driven predictive models for personalizing ion dynamics, scalable manufacturing platforms to overcome translational barriers, and rigorous biosafety protocols to ensure long-term safety and efficacy. The interdisciplinary integration of developmental biology, materials engineering, and multi-omics insights outlined here provides a robust foundation for metal ion-based precision therapies in nerve repair. Through continued interdisciplinary collaboration and a dedicated focus on translational medicine, metal ion-guided systems are positioned to advance a new generation of intelligent, adaptive, and patient-specific regenerative therapies with the potential to improve clinical outcomes.

## CRediT authorship contribution statement

**Mouyuan Sun:** Writing – original draft, Visualization, Validation, Methodology, Investigation, Funding acquisition, Formal analysis, Data curation, Conceptualization. **Yaxian Luo:** Writing – original draft, Validation, Methodology, Investigation, Formal analysis. **Zhixu He:** Writing – original draft, Validation, Methodology, Investigation. **Yan Tu:** Methodology. **Shuangyang Li:** Conceptualization. **Luying Qin:** Conceptualization. **Jingyu Zhang:** Conceptualization. **Lianjie Peng:** Formal analysis. **Tao Qiu:** Investigation. **Tian Zhang:** Formal analysis. **Huiming Wang:** Project administration. **Haifei Shi:** Writing – review & editing, Supervision, Project administration, Conceptualization. **Yong He:** Writing – review & editing, Supervision, Software, Project administration. **Mengfei Yu:** Writing – review & editing, Visualization, Supervision, Resources, Project administration, Methodology, Funding acquisition.

## Ethics approval and consent to participate

As this is a review article without direct human participants or samples, formal institutional review board (IRB) approval or written informed consent from individuals was not required.

## Declaration of competing interest

All authors declare no known competing financial interests or personal relationships that could have appeared to influence the work reported in this article.
