# Supplementary material for: From blueprint to build: Metal ions in peripheral nerve development and engineering regeneration
Source: Bioact Mater. 2026 Jun 9;64:915–49. doi: 10.1016/j.bioactmat.2026.05.019 (PMC13273508; doi:10.1016/j.bioactmat.2026.05.019)
Supplement: Multimedia component 1 [file mmc1.docx]

**Supporting Information of**

**From Blueprint to Build: Metal Ions in Peripheral Nerve Development and Engineering Regeneration**

1. Meta Analysis

This systematic review and network meta-analysis was registered with PROSPERO (CRD420251169618) and conducted in accordance with the PRISMA 2020 statement. A comprehensive literature search was performed independently by two investigators across four electronic databases—PubMed (MEDLINE), Embase, Web of Science, and Cochrane CENTRAL—from January 1, 1965, to October 1, 2025, with no language restrictions. Search terms including "digital nerve," "nerve injury," "nerve repair," "surgery," and related free-text and controlled vocabular were combined using Boolean operators. After a systematic screening process, 68 studies met the eligibility criteria. Methodologically, we constructed a connected evidence network and performed parameter estimation using Markov chain Monte Carlo (MCMC) algorithms, running four chains for 50,000 iterations with a 20,000-iteration burn-in period to ensure convergence, while employing random-effects models to account for between-study heterogeneity. Included studies involved patients with clinically or intraoperatively confirmed digital nerve injuries undergoing surgical repair, such as artificial conduit, nerve allograft, autologous vein graft, autologous nerve graft, or neurorrhaphy. Studies were included if they reported at least one sensory outcome (static two-point discrimination or modified Highet scale) at ≥6 months postoperatively. Eligible designs comprised prospective or retrospective cohort studies, case series (with sample size ≥1 and complete data), and randomized or non-randomized controlled trials. Exclusions comprised non-digital nerve injuries, conservative management, animal studies, reviews, conference abstracts, theses, duplicate publications, and studies with missing data or follow-up <6 months. A Bayesian network meta-analysis was performed using R software (version 4.3.0) with the gemtc and rjags packages, applying random-effects models to estimate relative effects and rank treatments.


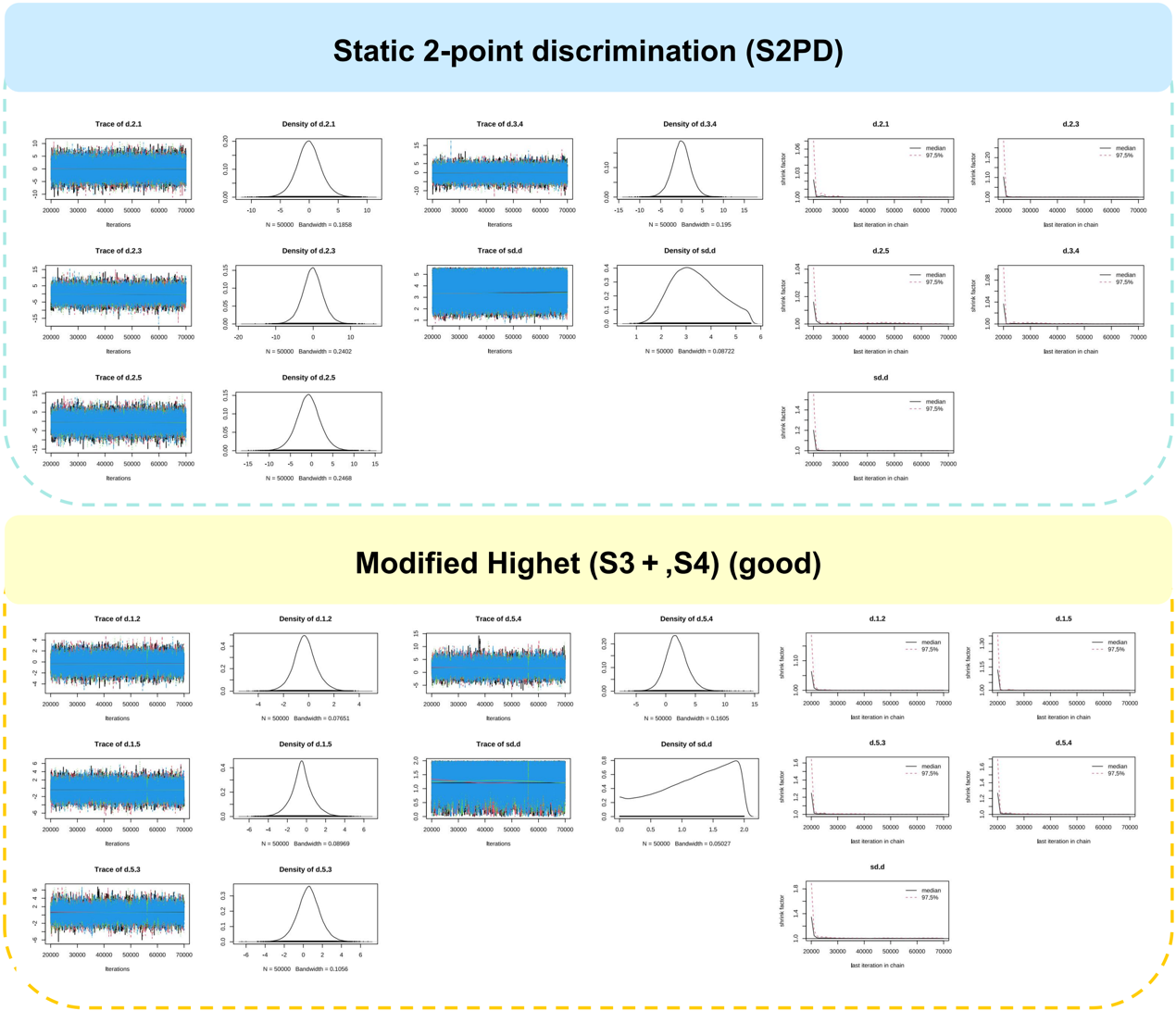


Supplementary Figure 1. Quality Assessment of Included Studies​.

1. Single-cell analysis

The single-cell data used in this study were downloaded from the datasets mentioned in the Supplementary table 1. Quality control was performed following the metrics from the original study.

The gene sets used in this study were downloaded from the GSEA database (https://www.msigdb.org/) mentioned in the Supplementary table 2.

Supplementary Table1

| Dataset source | Type | Species |
| --- | --- | --- |
| GEO database: GSE147285 | Transection injury (TI) | Mouse |
| GEO database: GSE216665 | Chronic crush injury (CCI) | Rat |
| GEO database: GSE265836 | Acute crush injury (ACI) | Rat |
| injured sciatic nerve atlas (iSNAT): https://cdb-rshiny.med.umich.edu/Giger_iSNAT/ | Crush injury (CI) | Mice |
| Sciatic Nerve ATlas (SNAT): https://www.snat.ethz.ch) | Development | Mice |

Supplementary Table2

| Standard name | Exact source |
| --- | --- |
| GOBP_CELLULAR_RESPONSE_TO_LITHIUM_ION | GO:0071285 |
| GOBP_CELLULAR_RESPONSE_TO_COPPER_ION | GO:0071280 |
| GOBP_CELLULAR_RESPONSE_TO_IRON_ION | GO:0071281 |
| GOBP_CELLULAR_RESPONSE_TO_CALCIUM_ION | GO:0071277 |
| GOBP_CELLULAR_RESPONSE_TO_MAGNESIUM_ION | GO:0071286 |
| GOBP_CELLULAR_RESPONSE_TO_ZINC_ION | GO:0071294 |


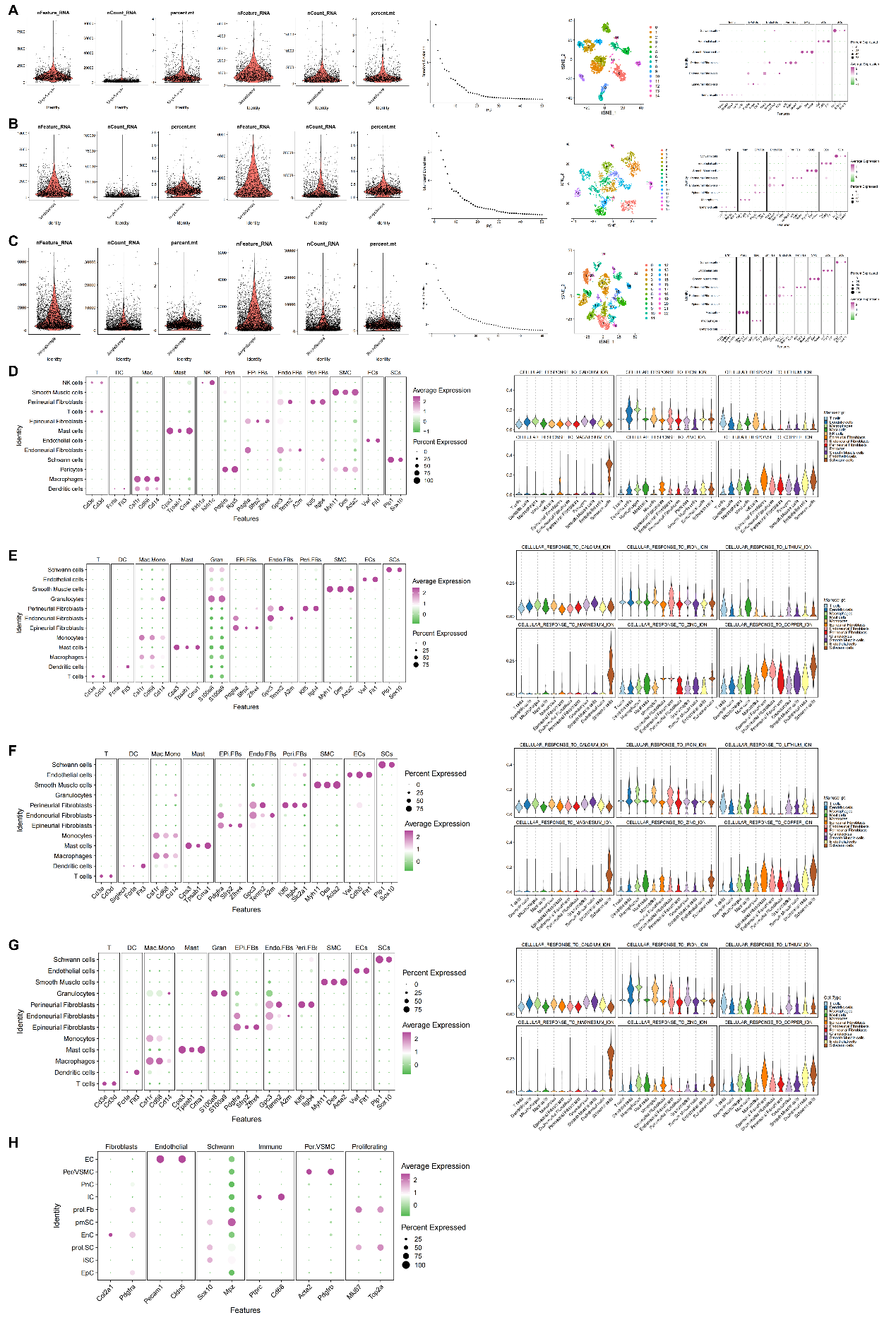


Supplementary Figure 2. Processing of mouse single-cell data.

(A-C). Quality control and cell clustering of mouse nerve transverse injury single-cell data.

(D-G) Cluster-defining markers and metallomic signature scoring in critical cell populations of mouse compression injury models.

(H) Cluster-defining markers of neonatal mice.


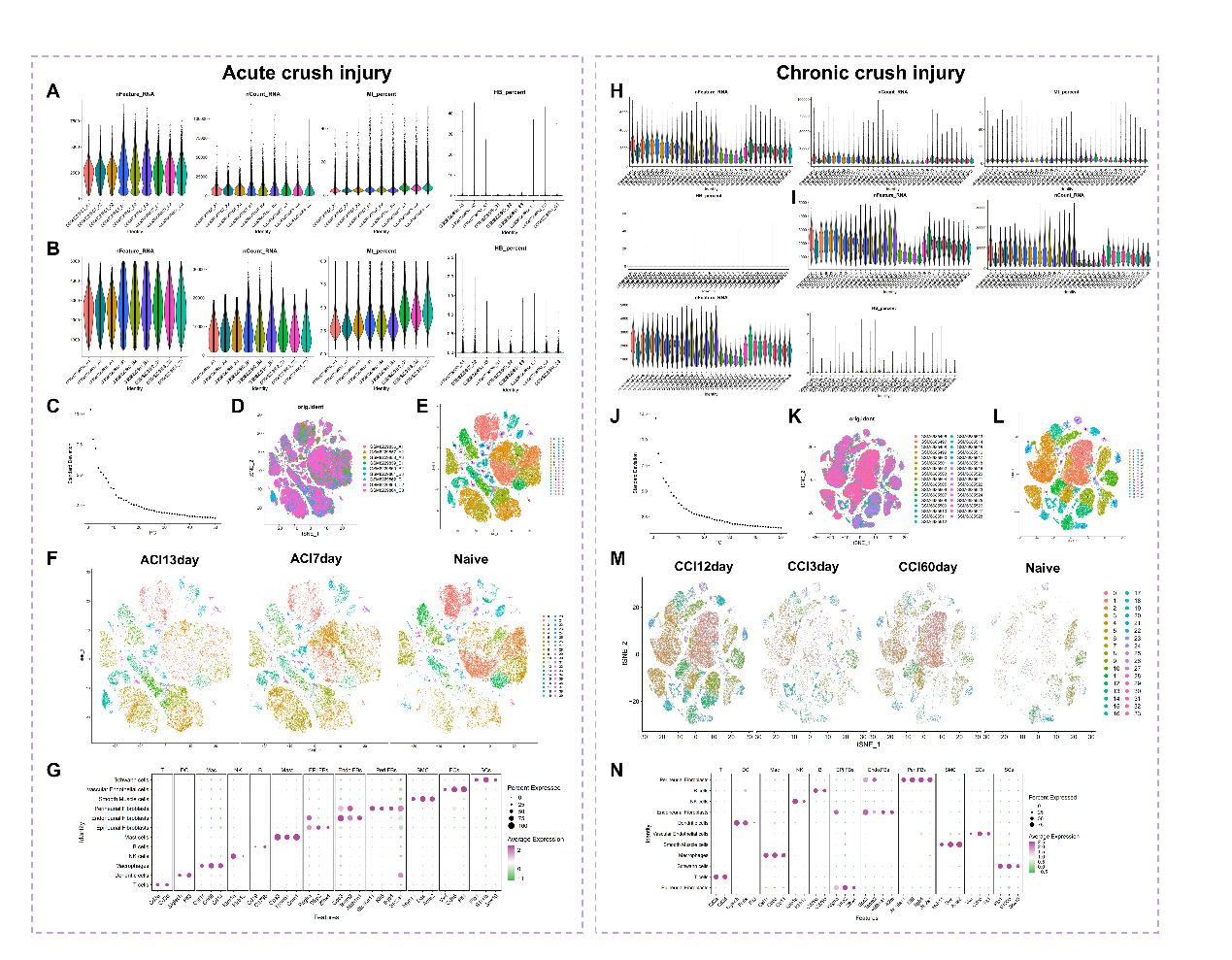


Supplementary figure 3. Processing of rat single-cell data.

(A-B) Quality control of rat nerve acute compression injury single-cell data.

(C-G) Single-cell clustering workflow in rat acute compression injury models.

(H-I) Quality control of rat nerve chronic compression injury single-cell data.

(J-N) Single-cell clustering workflow in rat chronic compression injury models.
